# Genome mining and metabolomics reveal context-dependent biosynthetic potential in a core poplar dark septate endophyte

**DOI:** 10.1128/msystems.00838-26

**Published:** 2026-09-23

**Authors:** Julian B. Cosner, Sameer Mudbhari, Jack A. Orebaugh, Robert L. Hettich, Paul E. Abraham, Tomás A. Rush

**Affiliations:** 1 Biosciences Division, Oak Ridge National Laboratory 6146, Oak Ridge, Tennessee, USA; 2 Bredesen Center for Interdisciplinary Research and Graduate Education, University of Tennessee 4292, Knoxville, Tennessee, USA; Wageningen University, Wageningen, the Netherlands; Wageningen University, Wageningen, the Netherlands

**Keywords:** dark septate endophytes, poplar, fungal metabolome, secondary metabolite, biosynthetic gene cluster, plant-microbe interactions

## Abstract

Dark septate endophytes (DSEs) are ubiquitous root-colonizing fungi characterized by melanized, septate hyphae and a frequent association with plants in stressful or nutrient-poor environments. Although DSEs are increasingly recognized for context-dependent effects on host stress resilience, the specialized metabolic capacity of many DSE lineages remains poorly resolved. *Hyaloscypha finlandica* is a recurrent root-associated fungus of *Populus* and other hosts, making it a useful system for understanding symbiotic mechanisms using multi-omics analyses. Here, we combined comparative biosynthetic gene cluster (BGC) prediction across 41 ascomycete genomes with untargeted metabolomics of *H. finlandica* PMI 746 grown in a defined minimal medium and a complex plant-derived medium. Benchmarking antiSMASH, FunBGCeX, and BGC-Prophet revealed broad conservation of PKS-, NRPS-, and terpene-associated biosynthetic capacity among DSEs. LC-MS/MS profiling detected strong medium-associated shifts in the extracellular chemical footprint of *H. finlandica* and identified putative terpenoid-, polyketide-, peptide-, siderophore-, and DHN-melanin-related features through spectral matching and *in silico* chemical-class inference. Authentic-standard comparison supported a probable assignment of 7-oxodehydroabietic acid, whereas most other annotations remain putative or class-level assignments. Integrating genomic and metabolomic evidence identified representative candidate intersections between encoded biosynthetic capacity and observed chemistry, but definitive BGC-product links remain targets for future genetic and structural validation. Together, these results provide a public paired-omics resource for a core *Populus*-associated DSE and a conservative framework for interpreting fungal multi-omics in non-model endophytes.

**IMPORTANCE:**

Dark septate endophytes (DSEs) are widespread fungal symbionts that influence plant performance, yet their encoded and expressed metabolic capacities remain largely uncharacterized. This study provides a paired comparative genome-mining and untargeted metabolomics resource for *Hyaloscypha finlandica*, a recurrent *Populus*-associated DSE. By explicitly distinguishing biosynthetic gene cluster (BGC) prediction, spectral annotation, authentic-standard support, and unresolved BGC-Prophet assignment, this work provides a transparent reference for future studies of DSE specialized metabolism. The resulting data sets support comparative analyses across fungal endophytes and help prioritize candidate metabolites and gene clusters for targeted validation.

## INTRODUCTION

Root-associated fungi are central to plant nutrition, stress tolerance, and ecosystem resilience. Beyond well-studied mycorrhizal symbionts, class IV endophytes, commonly referred to as dark septate endophytes (DSEs), are widespread but comparatively understudied root-associated fungi defined by melanized, septate hyphae and intraradical microsclerotia that occur across diverse plant hosts and ecosystems, particularly under stressful or nutrient-poor conditions (1–8). Despite their ubiquity, the mechanisms and chemical mediators underlying DSE-plant interactions remain poorly resolved. DSEs are frequently isolated from woody and herbaceous plants worldwide (9–11). Their recurrence across woody and herbaceous hosts, including communities dominated by ectomycorrhizal and arbuscular mycorrhizal fungi, indicates that DSEs represent a distinct component of plant root microbiomes (12–14).

Among DSEs, *Hyaloscypha finlandica* [(C.J.K. Wang & H.E. Wilcox) Vohnik, Fehrer & Réblová] (syn: *Phialophora finlandia, Cadophora finlandica*) has emerged as a broadly distributed and functionally relevant species (15–17). Although many *Hyaloscypha* species are described as saprobes of wood and leaf litter, *H. finlandica* frequently colonizes roots of ericoid, hardwood, and conifer hosts, where it can form mycorrhizal-like and endophytic associations (15, 17–24). Depending on the strain, host, and growth context, *H. finlandica* can have positive (10, 23, 25, 26) or negative (27) effects on plant growth. Recent time-series metabarcoding analyses suggest that *H. finlandica* is a recurrent and active root-associated fungus of *Populus tremuloides* across geographical gradients (14), further underscoring its ecological relevance as a DSE for multi-omics. Yet key biological mechanisms, including host specificity, colonization, metabolic activity, and nutrient exchange, remain unresolved for this and other DSE species (1, 3, 9).

Secondary metabolites (SMs) mediate competition, signaling, nutrient acquisition, and stress responses in plant-fungal interactions (28–31). Genome-scale mining enables automated detection and comparison of biosynthetic gene clusters (BGCs), offering a first-pass view of encoded biosynthetic potential (32). However, BGC prediction is not equivalent to production or product assignment. Most predicted fungal BGCs lack experimentally characterized products: in a large fungal genome atlas, only a small fraction of gene cluster families could be linked to known metabolites (33), and machine learning models remain constrained by limited validated training data despite estimates that Pezizomycotina alone may encode millions of secondary metabolites (34, 35).

Untargeted LC-MS/MS metabolomics provides a complementary view of expressed chemical output (36–39), but metabolite annotation is limited by incomplete spectral libraries, adduct/isomer ambiguity, and the inability of MS/MS alone to resolve many stereochemical and positional variants. As a result, genomics often predicts metabolites that remain chemically unobserved, while metabolomics detects compounds lacking genomic attribution. Recent advances in fragmentation modeling, molecular fingerprint prediction, and neural network-based classification, particularly within the SIRIUS framework, have expanded annotation coverage by enabling inference of molecular formulas, substructures, and chemical classes (40, 41). Complementary tools such as GNPS molecular networking and NPLinker help prioritize genome-metabolite links, but candidate links still require targeted validation (42–46).

Few studies have profiled metabolites directly from cultured DSE fungi, with most focusing instead on host responses or plant-facing chemistry (47–50). While some DSE taxa, such as *Periconia* spp., have genomic resources (51, 52), paired genome-mining and metabolomics data sets remain rare.

Motivated by the scarcity of DSE metabolomic references and the need to connect encoded biosynthetic potential with expressed chemistry, we addressed two questions: (i) do DSEs and closely related taxa exhibit consistent genomic capacity for secondary metabolism across lineages and literature-supported ecological categories, and (ii) does the extractable exometabolome of *H. finlandica* respond to defined culture media in ways consistent with this biosynthetic capacity? To answer these questions, we combined comparative genome mining across DSE and related ascomycetes with untargeted LC-MS/MS exometabolomics of *H. finlandica* PMI 746. We present this data set as a public paired-omics resource and as a conservative framework for prioritizing candidate BGC-metabolite relationships in non-model fungal endophytes.

## MATERIALS AND METHODS

### Fungal culture and growth conditions

*Hyaloscypha finlandica* strain PMI 746, originally isolated as a root endophyte from *Populus deltoides* (53), was used in all experiments. This strain was maintained as a 50% glycerol stock at −80°C and then subcultured on potato dextrose agar (PDA; BD Difco, New Jersey) prior to use. For initial plate cultures, 1.0 cm² mycelial fungal plugs were aseptically excised from the colony edge using a flame-sterilized cork borer and used to inoculate 10 new plates (100 × 15 mm) of PDA and 10 plates of glucose minimal medium (GMM) lacking thiamine (54). Plates were incubated at 25°C in the dark for 10 days. The resulting biomass was used for elemental profiling in Rush et al. (55). For liquid culture, fresh 1.0 cm² plugs from *H. finlandica* were excised from each respective medium and individually inoculated into 125-mL Erlenmeyer screw-cap flasks containing filled 50 mL of either potato dextrose broth (PDB; BD Difco, New Jersey) or GMM. Cultures were incubated in the dark at 25°C for 21 days at 250 rpm in an Eppendorf New Brunswick Excella E25 shaking incubator. PDB was used as a complex, plant-derived rich medium, whereas GMM was used as a defined minimal medium. For each medium, five biological replicates and five medium-only controls (no agar cores) were prepared. After incubation, cultures were vacuum-filtered through 0.45-µm membranes into sterile 50-mL Falcon tubes using a Millipore Steriflip vacuum system (SE1M003M00). Filtrates were stored at −80°C overnight and then freeze-dried (Labconco FreeZone 2.5 L Benchtop Freeze Dryer) with ventilated lids until completely dried.

### Metabolite extraction

Lyophilized fungal spent media were reconstituted in a 1:1 (vol/vol) mixture of HPLC-MS-grade water and ethyl acetate (1 mL each), vortexed, and phase-separated into two sterile vials. The aqueous extract was re-frozen and dried by lyophilization, while the ethyl acetate fraction was air-dried under sterile conditions. Dried aqueous extracts were resuspended in HPLC solvent A (98% water, 2% acetonitrile, and 0.1% formic acid), and dried ethyl acetate extracts were resuspended in HPLC solvent B (70% acetonitrile, 30% water, and 0.1% formic acid).

### Metabolite measurements and standard comparisons

Metabolomics data were acquired by liquid chromatography tandem mass spectrometry (LC-MS/MS) on a Vanquish ultra-high performance liquid chromatography (UHPLC) system (Thermo Fisher Scientific) coupled to a Q Exactive Plus mass spectrometer (Thermo Fisher Scientific) following procedures described previously (39). In brief, chromatographic separations were performed using a 75-µm inner diameter glass capillary packed in-house to 15 cm with Kinetex C18 beads (1.7 µm particle size; 100 Å pore size) using a 30-min gradient from aqueous to organic solvent (Solvent B increasing from 0% to 100% over 0–15 min, held at 100% from 15 to 16 min, then decreased to 0% from 16 to 18 min, and held at 0% until 30 min). Mass spectra were acquired in the positive ionization mode with full MS resolution of 70,000 and MS/MS resolution of 17,500 over an *m/z* range of 135–2,000. A data-dependent acquisition (DDA) method was used in which the top five most intense precursor ions per MS scan were selected for fragmentation, with dynamic exclusion of 10 s and stepped normalized collision energies of 10, 20, and 40 eV. Data were acquired using XCalibur v4.3 (Thermo Fisher Scientific). Full instrument setup and parameters are provided in Material S1.

Commercially available standards were purchased and measured for two prioritized metabolites: 1,3,6,8-tetrahydroxynaphthalene and 7-oxodehydroabietic acid. Standards were analyzed under the same LC-MS/MS platform and compared with *H. finlandica* features using precursor mass, retention behavior where available, and MS/MS mirror plots (Fig. S2). The 7-oxodehydroabietic acid standard produced a confident standard-supported MS/MS match to the corresponding *H. finlandica* feature. The 1,3,6,8-tetrahydroxynaphthalene comparison is reported in the supplement, but positional/isomeric ambiguity was not fully resolved by LC-MS/MS alone. We, therefore, refer to 7-oxodehydroabietic acid as a standard-supported probable annotation and retain conservative language for other features unless orthogonal structural validation is available.

### Metabolite data processing and annotation confidence

Raw LC-MS/MS files were processed in Compound Discoverer v3.3 (Thermo Fisher Scientific). Aqueous and organic extracts were analyzed separately because solvent-specific retention behavior prevents reliable cross-fraction feature alignment. For each fraction, 20 raw files (five biological replicates and five uninoculated controls per medium) were processed independently. The Compound Discoverer workflow included spectral selection, peak detection, feature grouping, compound annotation, and chemical composition prediction; full parameter settings are provided in Material S1. In parallel, molecular networking and *in silico* annotation were performed using GNPS2 (56) and SIRIUS v6.3 (57), respectively. Raw files were converted to .mzML format using MSConvert v3.0 with vendor peak picking (MS1-MS2). Classical molecular networking was run separately for aqueous and organic extracts in GNPS2 to generate consensus MS/MS spectra (.mgf), which were then used as input for SIRIUS.

SIRIUS outputs, including molecular formulae and CANOPUS-derived compound class predictions, were merged with the Compound Discoverer feature tables using rounded *m/z* values (three decimal places) and ion mass information. The structure_identification.tsv file was integrated in R v4.5.1 using a fuzzy join with an *m/z* tolerance of 0.001 Da to account for minor mass reporting differences. For features annotated by spectral matching to the NIST 2023 and GNPS libraries, chemical classification was assigned using InChIKeys for the closest retrievable compounds in PubChem or ChemSpider, followed by batch classification with ClassyFire (58). Spectral matches were retained only when (i) mass deviation was consistent with common adducts or analogs within ±60 Da and (ii) the NIST HighChem HighRes score was ≥70 and represented the top-ranked match for that feature. Annotation confidence was assigned using metabolomics reporting conventions derived from the Metabolomics Standards Initiative and subsequent small-molecule annotation frameworks (59, 60). Features supported by authentic-standard LC-MS/MS comparison are reported as standard-supported probable annotations; library matches without standards are reported as putative annotations; SIRIUS/CANOPUS and ClassyFire outputs are reported as formula-, substructure-, or class-level annotations; and unannotated features are retained as molecular features. We avoid treating library or *in silico* annotations as confirmed structures.

### Metabolomics statistical analysis

Untargeted metabolomics data from *H. finlandica* aqueous and ethyl acetate extracts were processed separately using a standardized statistical workflow in R v4.5.1 with tidyverse, vegan, rstatix, ggpubr, ggfortify, and reshape2. Peak area matrices and metadata exported from Compound Discoverer were condensed using an in-house script (https://github.com/dlcarper/Metabolite-Condensing-Script) to group features within a 0.05 Da *m/z* tolerance. Zero values were converted to NA, features containing only missing values were removed, and intensities were log2 transformed. Signal normalization was performed in two stages. First, LOESS normalization was applied separately within each set of biological replicates. The first replicate within each group was used as the reference, and each remaining replicate was normalized against the reference using LOESS fit across features, where differences in log2 abundance between the replicate and reference were modeled as a function of feature intensity. The fitted correction was applied to the replicate abundances. Because normalization was performed independently within each experimental group, samples from different biological conditions were not used to fit the same LOESS model. Second, features were median-centered across samples to remove remaining global offsets from differences in biomass production and injection load. Sample-wise intensity distributions were inspected to confirm comparable signal ranges across runs. Missing values were imputed under a left-censored, missing-not-at-random assumption using a downshifted Gaussian distribution (width = 0.3; downshift = 2.4; seed = 100).

Multivariate structure was assessed using Bray-Curtis dissimilarities calculated from the normalized, imputed feature matrix. Non-metric multidimensional scaling (NMDS) was performed with the metaMDS algorithm using multiple random starts to ensure convergence. Ordination scores were merged with sample metadata and visualized using group-specific colors and shapes with 95% confidence ellipses. For univariate analysis, each feature was modeled independently as a function of the experimental group using normalized, imputed log2 intensities. Linear models of the form intensity ~group were fit, and one-way analysis of variance (ANOVA) was used to test for overall group effects. *P*-values were adjusted using the Benjamini-Hochberg procedure; features with FDR-adjusted *P* < 0.05 were considered significant. Tukey HSD *post hoc* tests were applied to significant features to resolve pairwise contrasts. For effect size interpretation, log2 intensities were back-transformed to raw-scale abundances, and fold changes were calculated as ratios of group means.

Class-level differences between PDB and GMM conditions were quantified using mixed-effect models. Processed log2-transformed intensities were imported from a curated data set, and metabolite-specific abundances were modeled as value ~group + (1 | metabolite). Classes represented by a single metabolite were analyzed using fixed-effect models. Estimated marginal means and contrasts (PDB − GMM) were computed with emmeans. Log2 fold change estimates, 95% confidence intervals, and *P*-values were adjusted for multiple testing using Benjamini-Hochberg correction. For visualization, each class was annotated with the number of contributing metabolites, and the most abundant metabolite per class (highest mean log2 intensity) was labeled at the corresponding point estimate in forest plots.

### Fungal genome data set acquisition

A comparative genome-mining approach was used to assess secondary metabolic potential across DSE fungi and related taxa and to identify conserved BGC features. Based on published surveys of DSE diversity (3, 51, 61–63), we curated a data set representing described DSE species and closely related ascomycetes, including melanized and non-melanized outgroups spanning diverse ecological lifestyles (e.g., mycorrhizal symbionts, endophytes, saprotrophs, and opportunistic pathogens). Ecological categories were literature-derived, strain-/source-associated annotations based on primary literature and genome metadata, not fixed organismal identities. These categories were used for exploratory comparisons, recognizing that fungal lifestyles can be context-dependent and may shift with the host, substrate, developmental stage, and environment.

A total of 41 ascomycete genomes were retrieved from NCBI GenBank, including 19 DSE genomes and 22 ecological outgroups. For each assembly, the genomic FASTA, annotation (.gff), and predicted protein sequences (.faa) were downloaded using the NCBI data sets command-line tool. Species names were validated against Index Fungorum (*indexfungorum*.org) and Mycobank (*mycobank*.org), and genome accession numbers with ecological annotations and supporting sources are provided in Material S2.

### Mining biosynthetic potential

BGCs were identified using three complementary genome-mining pipelines representing distinct methodological paradigms: antiSMASH (rule based) (64), FunBGCeX (profile/HMM based) (65), and BGC-Prophet (deep learning based) (66). All tools were run with default or recommended parameters. Tool outputs were interpreted as predicted biosynthetic capacity and reference-cluster similarity, not direct product identification.

antiSMASH v8.0.1 was executed via the fungal web server, with the detection strictness set to relaxed to retain partial or atypical clusters (e.g., NRPS-like). For each genome, antiSMASH reported predicted BGC regions with genomic coordinates, cluster class assignments (e.g., PKS, NRPS, RiPP, and terpene), hybrid designations, and core biosynthetic genes. KnownClusterBlast was used to compare predicted clusters to the MIBiG v4.0 database, and the most similar reference cluster and confidence level were recorded as reference similarities. Cluster counts and annotations were extracted from GenBank outputs.

FunBGCeX v1.0.0 was run following the authors’ published workflow. The pipeline identifies biosynthetic genes of interest, including canonical core enzymes and domainless biosynthetic proteins, and evaluates flanking genomic regions for additional biosynthetic signatures using an HMMER v3.4 library and DIAMOND v2.2.0 searches against a curated BGC protein database. Regions meeting predefined distance and gene-content criteria and colocalized with core biosynthetic genes were reported as BGCs.

BGC-Prophet v0.1.0 was installed in a Conda environment and ran in full mode using pretrained detection and classification models (annotator.pt and classifier.pt). Predicted protein sequences (.faa) were used as input. The pipeline embeds protein sequences using the ESM2 language model, predicts BGC-associated genes at a probability threshold of 0.5, merges adjacent predictions into clusters (maximum gap of three genes; minimum cluster size of two genes), and assigns product classes. Output CSV files reported predicted BGCs with confidence scores and annotations.

### Genome mining analysis

BGC predictions from all tools were parsed and compiled into tool-specific tables containing a unique BGC identifier, species name, ecological category, region designation (for antiSMASH), cluster class, genomic coordinates, and associated reference-product information. Genome and proteome assemblies were evaluated using BUSCO v6.0.0 with the ascomycota_odb12 lineage data set (2,826 single-copy orthologs). Only genomes with ≥70% complete BUSCO scores were retained. A taxonomic reference tree was generated using the NCBITaxa ete3 v3.1.3 function based on NCBI TaxIDs for all 41 fungi. Hierarchical relationships were retrieved, strain-level leaves were duplicated as needed, and the resulting topology exported in Newick format (.nwk) for visualization in iTOL v7.2.2.

BGC predictions were aggregated across genomes to compare per-genome cluster counts and class composition by tool and ecological category. Statistical comparisons among ecological groups were conducted using ANOVA with pairwise *post hoc* tests, alongside nonparametric Wilcoxon tests with Benjamini-Hochberg correction within the tool. Phylogenetically informed regressions were performed using the phylolm v2.6.5 package in R under a lambda model, equivalent to PGLS when the same lambda is applied. Analyses were repeated using both the NCBI taxonomy-based tree and a genome-derived MashTree v1.4.6 phylogeny (67) to assess sensitivity to tree choice.

For antiSMASH outputs, GenBank files were parsed with Biopython to extract cluster classes and genomic coordinates. Custom Python scripts assigned clusters to canonicalized classes (NRPS, PKS, RiPP, terpene, indole, saccharide, alkaloid, hybrid, other, and unknown), retaining original annotations for traceability. Regions with multiple or class-like assignments were classified as hybrids. Genome sizes were calculated from assembled FASTA files and included as reference metadata. All genome-mining data processing and statistical analyses were performed using custom Python v3.12.3 and R v4.4.2 scripts, with visualization in ggplot2. To avoid equating raw BGC counts with biosynthetic diversity, predicted clusters from antiSMASH were additionally grouped into gene cluster families (GCFs) using the sequence-similarity and shared-biosynthetic-gene context across predicted regions using BiG-SCAPE v2.0.3. As this genome mining approach was exploratory rather than confirmatory, candidate *H. finlandica* clusters corresponding to prioritized metabolite annotations were not inspected for domain architecture or synteny relative to MIBiG or literature reference clusters. Because fungal clusters with similar domain architecture can produce distinct products, BGC totals and GCF membership were used to assess relatedness and candidate plausibility rather than to claim product identity.

## RESULTS

### Comparative biosynthetic potential among DSEs and related ascomycetes

**Fig 1:**
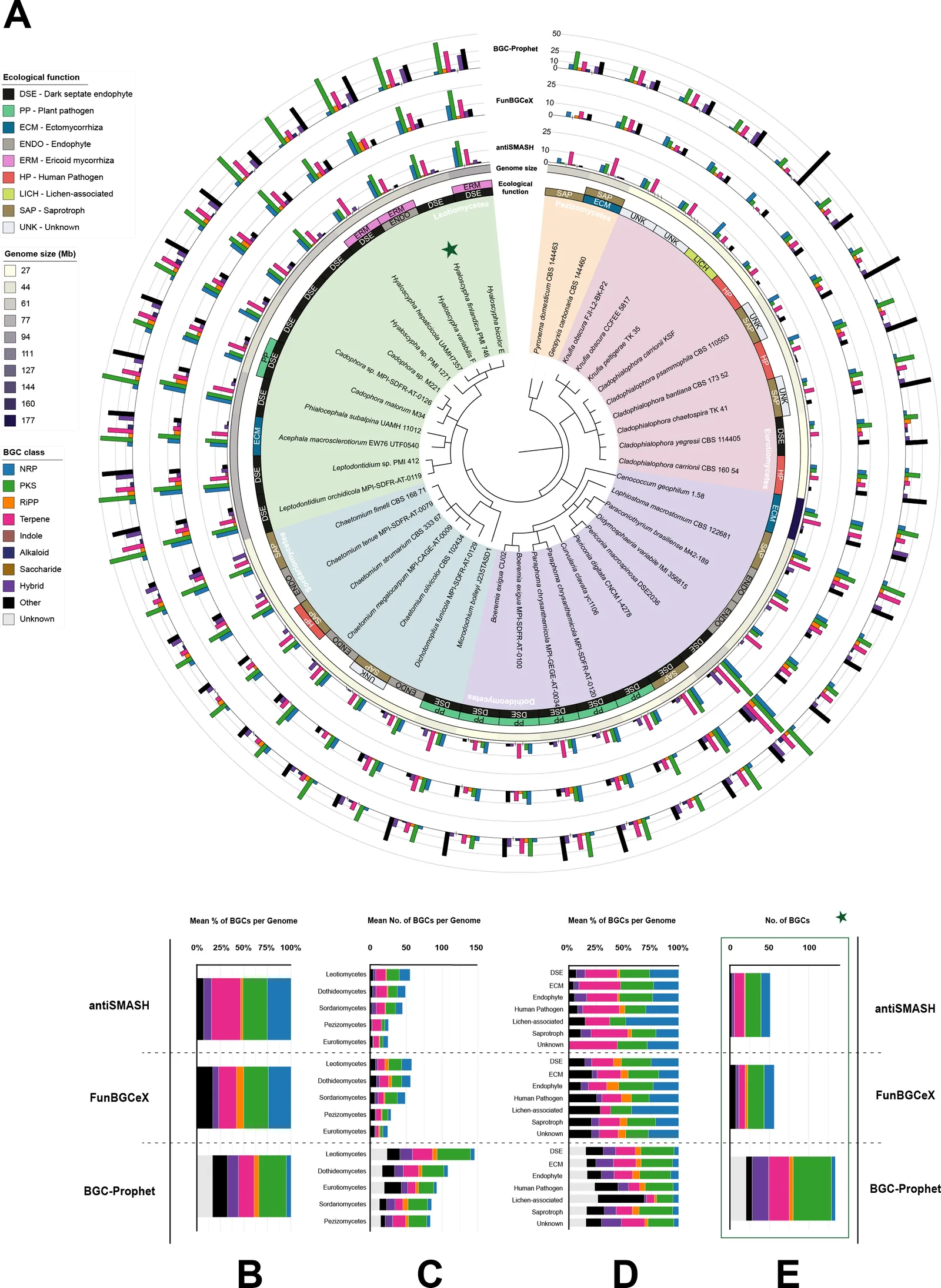
Comparative genome mining and biosynthetic potential across 41 ascomycete fungi and related taxa. (**A**) The genome data set of 41 ascomycete and closely related taxa was visualized in the Interactive Tree of Life (iTOL) using an NCBI taxonomy-based tree (.nwk). The phylogeny was annotated with 10 canonicalized biosynthetic gene cluster (BGC) classes—NRPS, PKS, RiPP, terpene, indole, alkaloid, saccharide, hybrid, other, and unknown. Ecological metadata (i.e., inner to outer ring), including fungal class (outer shaded background) and strain-specific source-published ecology (primary and secondary assignments), are displayed as the first and second concentric rings. The third ring represents genome size (Mb), scaled from light (small) to dark (large). Rings 4–6 show per-genome BGC counts by class predicted by antiSMASH, FunBGCeX, and BGC-Prophet, respectively. (**B**) Mean composition (%) of BGCs per genome by prediction tool, displayed as stacked bars normalized by total predicted clusters per genome. (**C**) Mean number of predicted BGCs per genome by fungal class and tool, showing lineage-specific enrichment of polyketide (PKS) and peptide (NRPS) clusters. (**D**) Mean composition of BGCs per genome by ecological function and tool. (**E**) Genome-specific BGC-class profiles for *Hyaloscypha finlandica* PMI 746, denoted by the green box/star in panel **A**.

Using antiSMASH, FunBGCeX, and BGC-Prophet, we observed tool-specific differences in both the number and class composition of predicted BGCs, yet strong rank concordance was observed across 41 sampled ascomycete genomes (Fig. 1A). Across the data sets, the three approaches predicted a total of 1,715 (antiSMASH), 1,886 (FunBGCeX), and 4,524 (BGC-Prophet) BGCs, corresponding to means of 41.8, 46.0, and 110.3 clusters per genome, respectively. Genome-level totals were highly correlated between antiSMASH and FunBGCeX (Spearman ρ = 0.964, *P* < 4e−24) and moderately correlated between each of those tools and BGC-Prophet (ρ = 0.645, *P* < 5e−6; ρ = 0.573, *P* < 9e−5), respectively. Genome size was positively associated with BGC counts for all tools: antiSMASH (ρ = 0.60, *P* < 3e−5); FunBGCeX (ρ = 0.54, *P* < 2e−4); and BGC-Prophet (ρ = 0.82, *P* < 4e−11). These results indicate that larger genomes generally encode more predicted clusters. Class composition was method dependent: antiSMASH totals were enriched for terpenes (30.4%), PKS (29.2%), and NRPS (26.1%); FunBGCeX totals were enriched for PKS (28.5%), NRPS (23.9%), and terpenes (19.2%); and BGC-Prophet totals were enriched for PKS (29.4%), terpenes (17.3%), unknown (16.5%), other (12.5%), and hybrid (11.6%) (Fig. 1B).

**Fig 2:**
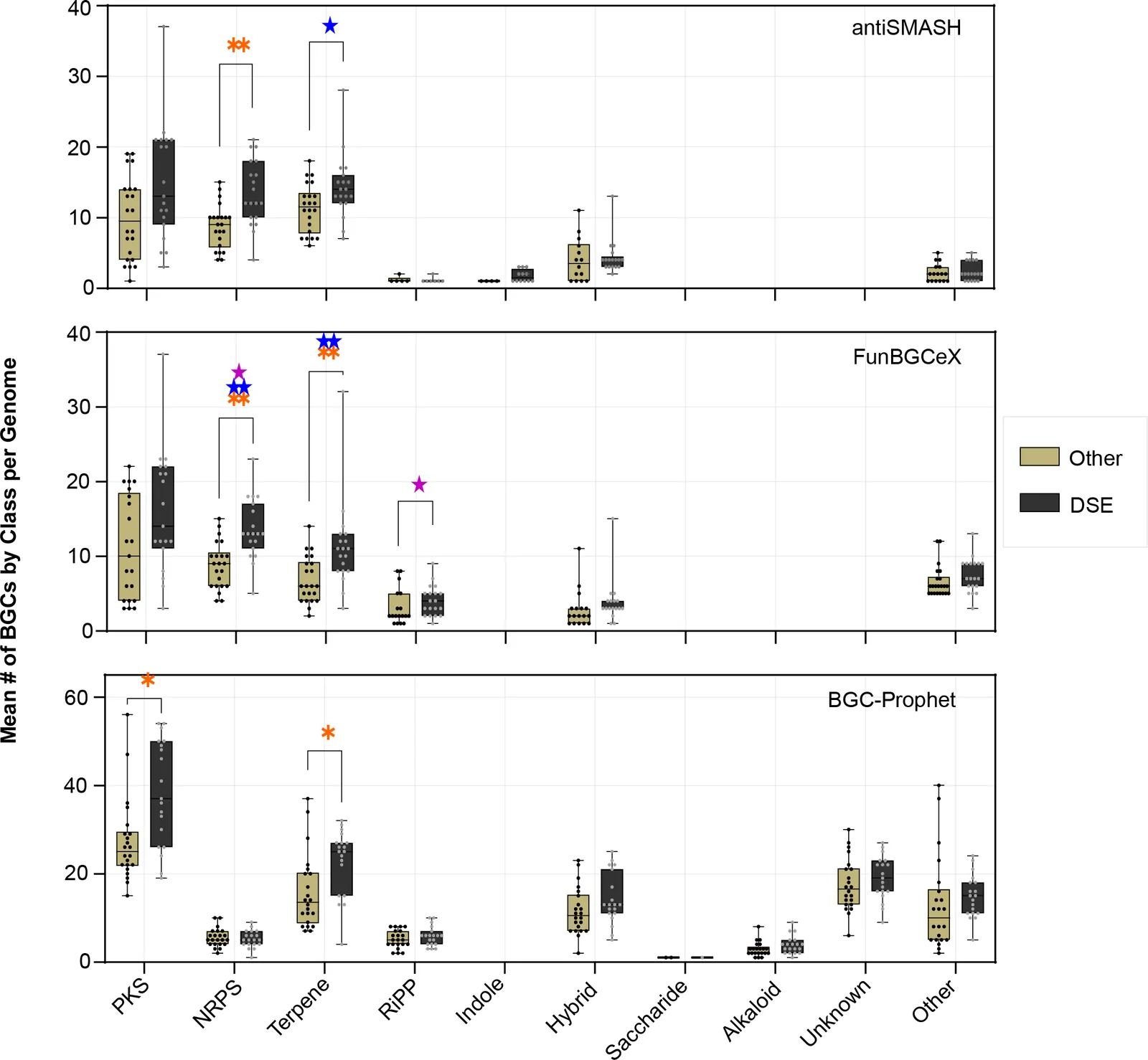
Genomes of dark septate endophytes (DSE) encode for some biosynthetic gene cluster (BGC) classes more than other related ascomycetes across three mining tools. Stacked box-and-whisker plots per tool show the distribution of per-genome BGC counts for each predicted class in other (light; *n* = 22) and DSE (dark; *n* = 19) fungi. Brackets denote DSE versus other comparisons, and significance is represented by single (*P* < 0.05) or double (*P* < 0.01) symbols: Wilcoxon (Benjamini-Hochberg correction within tool [orange asterisks]); PGLS (phylogeny-aware [blue star]); PGLS (taxonomy-aware [magenta star]). These statistics are presented as exploratory within a reference data set; differences across tools reflect methodological and model-awareness variation rather than guild representation or a benchmark of tool performance.

Leotiomycetes and Dothideomycetes had the highest mean per-genome BGC counts (Fig. 1C). Literature-derived ecological grouping showed similar trends: DSE genomes harbored higher NRPS and terpene cluster counts for several tools, whereas differences in PKS and fungal RiPP counts were inconsistent and often tool dependent (Fig. 1D and 2).

### Enrichment of terpene-, PKS-, and NRPS-associated BGCs in DSE genomes

After Benjamini-Hochberg adjustment, DSE genomes had significantly more NRPS counts in antiSMASH (*q* = 0.0093; means: 13.5 vs 8.7), while the terpene difference in antiSMASH was not significant (*q* = 0.075) (Fig. 2). In FunBGCeX, NRPS (*q* = 0.0018) and terpene (*q* = 0.0070) counts were higher in DSEs; PKS, RiPP, and hybrid counts were not significant after correction. BGC-Prophet predicted enrichment for PKS (*q* = 0.0188) and terpenes (*q* = 0.0188) in DSEs (means: PKS, 38.4 vs 27.3; terpenes, 22.8 vs 15.8). To account for shared ancestry, we used phylogenetic generalized least-squares (PGLS) models with both taxonomy-based and genome-derived trees. Of 60 planned PGLS models (three tools × 10 BGC classes × two trees), 42 (70%) had sufficient variation and were successfully fitted, including all 30 models spanning the five major classes (PKS, NRPS, terpene, RiPP, and hybrid), whereas the remaining 18 involved sparse classes with insufficient variation. DSE enrichment persisted for NRPS and terpenes in the genome-derived models (FunBGCeX NRPS *P* = 0.0099; FunBGCeX terpenes *P* = 0.0071; antiSMASH terpenes *P* = 0.038), and the taxonomy tree similarly supported NRPS enrichment (FunBGCeX NRPS *P* = 0.0469) while also supporting higher RiPP counts in DSEs (FunBGCeX RiPP *P* = 0.0264). At the gene-cluster family level, BiG-SCAPE grouped 1,715 antiSMASH regions into 1,245 GCFs, most of which were single-genome singletons (*n* = 989). DSE genomes had higher total GCF counts than other genomes (*q* = 0.0196; means: 50.2 vs 34.5), including higher NRPS GCF counts (*q* = 0.0196; means: 13.9 vs 9.3). These family-level differences were not significant after phylogenetic correction (NRPS PGLS *P* = 0.248 with the taxonomy tree and *P* = 0.0895 with the genome-derived tree), indicating that the GCF signal supports the BGC count patterns but remains partly confounded with phylogenetic structure.

### Reference product labels differ among genome-mining tools

**Fig 3:**
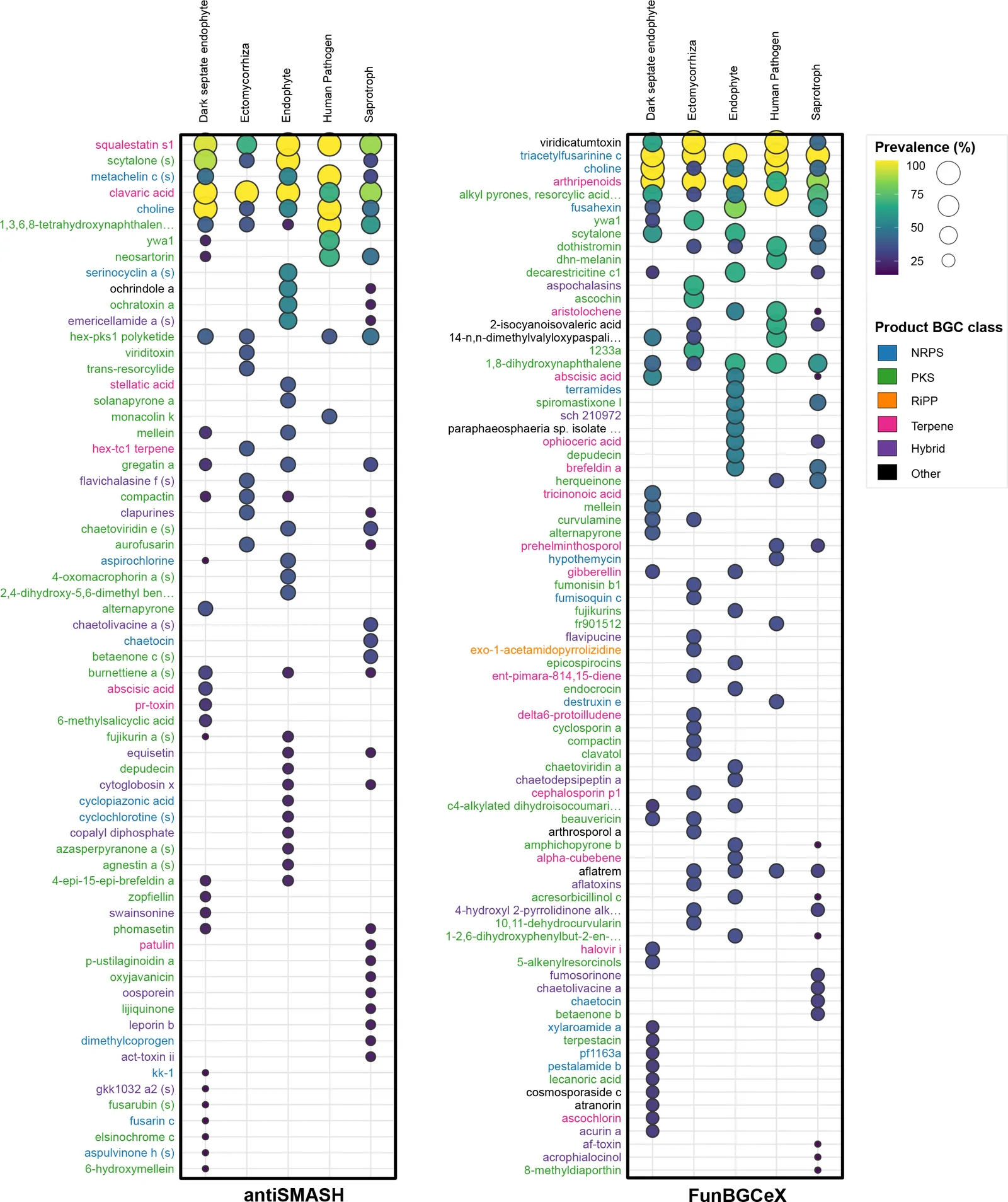
Comparative distribution of predicted biosynthetic products across ecological guilds reveals conserved repertoires by genome-mining tools. antiSMASH and FunBGCeX predictions of “Most similar” secondary metabolites are shown as independent bubble plots to illustrate tool-specific ecological patterns. Each panel depicts the top 30 (top to bottom) putative compounds per ecological guild following exclusion of guilds represented by fewer than three genomes per tool. Bubble diameter and color gradient correspond to the proportion of genomes within each guild that contain ≥ 1 biosynthetic gene cluster (BGC) assigned to that compound. Compound name color corresponds to predicted BGC class (NRPS, PKS, RiPP, terpene, hybrid, and other). Compound names were standardized using a canonicalization pipeline that removed stereochemical prefixes, punctuation inconsistencies, and applied both a manual synonym map [e.g., (R)-mellein, (−)-mellein, mellein derivative = mellein] and fuzzy string matching (Jaro-Winkler ≤ 0.10) to collapse near-identical annotations. Where multiple candidate products were listed for a single cluster (e.g., “compound A/compound B”), only the first was retained and denoted with the suffix “(S)” to indicate a putative set of analogs. To improve readability, names exceeding 30 characters were truncated with an ellipsis (“…”). Analyses were performed independently for each tool to avoid cross-platform bias in annotation vocabulary. Therefore, *Y*-axes differ between panels and reflect the most prevalent products from reference-based matching predicted by each pipeline.

Predicted reference product labels differed widely between antiSMASH (*n* = 115) and FunBGCeX (*n* = 286) with relatively few exact annotation overlaps (*n* = 31). Even among shared labels, genome-level agreement was modest (Jaccard index = 0.41; frequency-weighted = 0.46), with only six compounds showing identical co-prediction. These patterns indicate that the tools converge more strongly on relative biosynthetic capacity and broad BGC class than on specific reference-product labels. We therefore treat named labels such as squalestatin, resorcylic acid, arthripenoids, clavaric acid, triacetylfusarinine, and 1,3,6,8-tetrahydroxynaphthalene as reference similarities from genome-mining databases and not as direct evidence that the same compounds are produced by the queried fungi (Fig. 3). Few shared labels (e.g., abscisic acid, YWA1, and choline) were observed and were primarily associated with conserved cluster families rather than direct tool-specific product agreement.

### Predicted metabolic repertoire of *H. finlandica*

All three tools converged on a large and diverse predicted BGC repertoire for *H. finlandica* (Fig. 1E). The genome encoded multiple NRPS, PKS, and terpene-class clusters, with BGC-Prophet contributing substantial numbers of Unknown and Hybrid designations. antiSMASH generated several high-confidence reference similarities (e.g., clavaric acid-, YWA1-, PR-toxin-, and 1,3,6,8-tetrahydroxynaphthalene-like regions), whereas FunBGCeX produced a wider array of reference similarities including arthripenoid-, YWA1-, ascochlorin-, atranorin-, and abscisic acid-like regions. Notably, FunBGCeX reported both a cosmosporaside C-like transferase region found exclusively in Helotiales DSEs within the data set and a hydroxyferricrocin-like NRPS region in *H. finlandica* with confident reference similarities (score >70).

### Exometabolomic profiling of *H*. *finlandica* spent media

**Fig 4:**
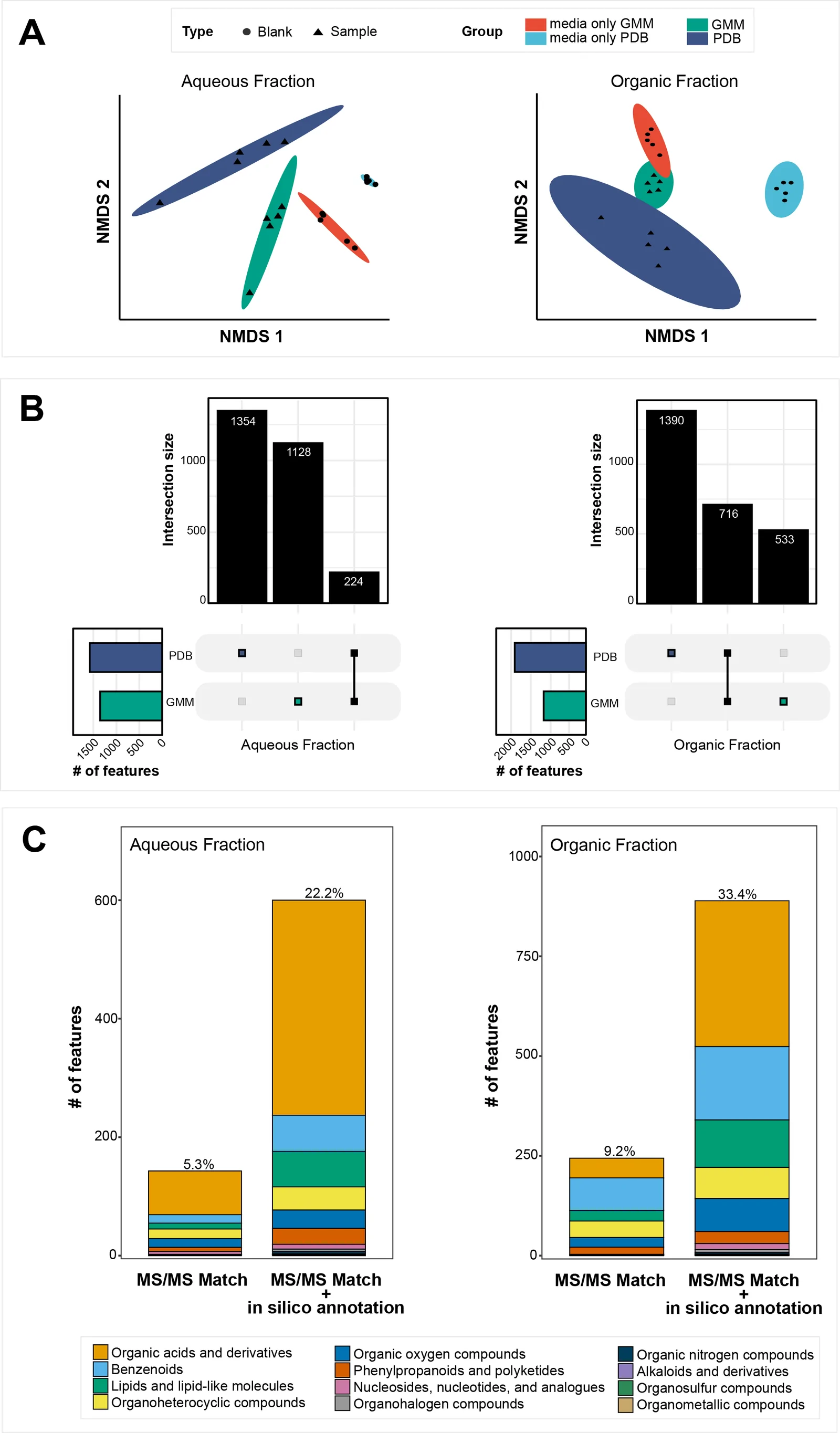
Chemotaxonomic profile measured from *H. finlandica* PMI 746 exudates using untargeted metabolomics. (**A**) Nonmetric multidimensional scaling (NMDS) ordinations of aqueous (left) and organic (right) LC-MS/MS feature matrices showing separation among *Hyaloscypha finlandica* PMI 746 cultures grown in glucose minimal medium (GMM) or potato dextrose broth (PDB) and their corresponding uninoculated medium controls. Ellipses represent 95% confidence intervals around group centroids. (**B**) Numbers of LC-MS/MS features significantly enriched in fungal exudates relative to matched uninoculated media for aqueous and organic extracts. Features were identified by one-way ANOVA followed by Tukey HSD (FDR < 0.05, log2 fold change ≥ 2). (**C**) Chemical superclass composition of enriched features based on structural annotation. Putative annotations were assigned via spectral library matching (NIST 2023 and GNPS) and extended using SIRIUS/CANOPUS predictions, enabling classification to the ClassyFire superclass level. Stacked bars indicate the relative contribution of major chemical superclasses among annotated aqueous and organic exudate features.

Untargeted metabolomics of *H. finlandica* spent media detected 33,777 aqueous and 26,897 organic features with quantifiable signal across cultures and medium controls. These features, defined as ions observed at least once across all samples, formed the basis for downstream statistical and annotation analyses. Nonmetric multidimensional scaling (NMDS) revealed clear separation among GMM, PDB, and their respective medium controls in both fractions (Fig. 4A), indicating strong effects of nutrient context and fungal activity on metabolite profiles.

Feature-wise one-way ANOVA with Benjamini-Hochberg correction identified 9,197 aqueous (27.2%) and 5,238 organic (19.5%) features with significant group effects (FDR < 0.05). These signals likely represent a mixture of fungal-derived metabolites, fungal-modified medium constituents, lysed cell products, and other condition-dependent compounds. To distinguish fungus-associated metabolites, cultures were compared directly to matched medium blanks (Fig. 4B). In the aqueous extract, 1,351 features were enriched in GMM-derived exudates and 1,578 in PDB-derived exudates relative to their respective blanks (Tukey HSD <0.05; log2 fold change ≥2). In the organic extract, 1,248 features were enriched in GMM cultures and 2,106 in PDB cultures. Across both fractions, this yielded 2,705 nonredundant aqueous and 2,638 nonredundant organic features enriched in at least one fungal-versus-blank contrast, representing candidates for *H. finlandica*-associated extracellular metabolite features.

Structural annotation was achieved through a combination of spectral library matching and *in silico* prediction. Filtered spectral matches (HighChem HighRes ≥ 70, ±60 Da analog/adduct allowance) yielded putative identities for 5.9% of aqueous and 9.2% of organic features using NIST 2023 and GNPS libraries, respectively. To extend coverage, SIRIUS-based predictions enabled 22.2% of aqueous and 33.4% of organic features to be assigned to at least the ClassyFire superclass level (Fig. 4C). These values reflect annotation coverage, not confirmed structural identification.

Chemical classification revealed a broad and diverse metabolite repertoire (Table S1). Carboxylic acids and derivatives dominated the annotated space, driven largely by amino acids, peptides, and related analogs. Fatty acyls and other primary metabolism-associated classes were also abundant. Multiple features corresponded to secondary metabolite classes or precursors, including linoleic acid derivatives and sesquiterpenoid-like annotations, consistent with genome-predicted biosynthetic capacity for PKS-, NRPS-, and terpene-associated biosynthesis.

### Authentic-standard and representative metabolite annotation support

Authentic-standard comparisons strengthened annotation confidence for one prioritized metabolite. The 7-oxodehydroabietic acid standard produced a close MS/MS mirror match to the *H. finlandica* feature and is, therefore, reported as a standard-supported probable annotation (Fig. S2). Because no single syntenic terpene BGC could be assigned to this feature, it is not treated as a resolved BGC product. The 1,3,6,8-tetrahydroxynaphthalene standard comparison is also reported in the supplement, but the observed *H. finlandica* dihydroxynaphthalene-like feature could not be resolved to a unique positional isomer using LC-MS/MS alone. We, therefore, describe DHN-melanin-related features conservatively as putative aromatic intermediates. Similarly, library-based annotations such as desmethyldiaportinol, brefeldin C-like features, desferricrocin-/ferricrocin-like features, glycerophosphocholine, resorcinol-O, O-diacetic acid-like features, and caryophyllene oxide remain putative without independent support by standard or orthogonal structural evidence.

### Integrating genome-mining predictions with metabolite annotations

Direct alignment between genome mining predictions and untargeted metabolomics remains challenging because the two approaches define chemical similarity using fundamentally different criteria. Genome mining tools report reference similarities based on biosynthetic homology, core-gene class, domain architecture, and local gene content, whereas metabolomics annotations rely on precursor mass, fragmentation similarity, substructure inference, and library-specific nomenclature. As a result, predicted BGC product labels and detected metabolites often lack shared naming vocabularies, even when they derive from related chemical scaffolds.

To address this mismatch, we harmonized predicted BGC product labels with LC-MS/MS annotations using a canonicalization and manual curation workflow that normalized compound names, collapsed synonyms, and incorporated substructure-level classifications. This approach revealed several representative intersections between encoded capacity and detected features, including DHN-melanin-related intermediates consistent with PKS/T1PKS predictions, siderophore-associated NRPS products matching ferricrocin/desferricrocin-like annotations, aromatic PKS/T3PKS-like chemistry, and a low-confidence brefeldin-like candidate intersection (Table 1). These correspondences do not constitute definitive structure-BGC assignments; rather, they identify high-priority candidates where genomic potential and metabolomic evidence converge.

**TABLE 1:** Representative similarities between predicted biosynthetic gene clusters (BGCs) and LC-MS/MS compound annotations in *Hyaloscypha finlandica* PMI 746^*e*^

Tool	BGC class	Genomic prediction top candidate	Tool confidence score	Metabolomic prediction top candidate	Spectral match confidence score^*a*^
antiSMASH	PKS/T1PKS	1,3,6,8-Tetrahydroxynaphthalene	High	Dihydroxynaphthalene^*c*^	88
antiSMASH	PKS/T1PKS	4-Epi-15-epi-brefeldin A	Low	Brefeldin C	78
antiSMASH^*b*^	NRPS	Triacetylfusarinine C or hydroxyferricrocin	High	Desferricrocin	79
FunBGCeX	NRPS or NRPS-like	Choline	High	Glycerophosphocholine	92
FunBGCeX	PKS/T3PKS	Alkyl pyrones, resorcylic acids, and resorcinols	Med	Resorcinol-O,O′-diacetic acid^*d*^	77

^*a*^ Spectral match confidence using NIST 2023 HighChem HighRes score (low = 0; high = 100).

^*b*^ This compound was also identified by the FunBGCeX tool.

^*c*^ The positional isomers of the two hydroxyl groups could not be resolved using LC-MS/MS.

^*d*^ The detected putative compound corresponds to the dehydrated protonated ion [M + H − H₂O].

^*e*^ Putative compound-level intersections identified by reference-based genome mining tools and matched to spectral libraries are shown.

### Medium-dependent metabolite responses of *H. finlandica*

**Fig 5:**
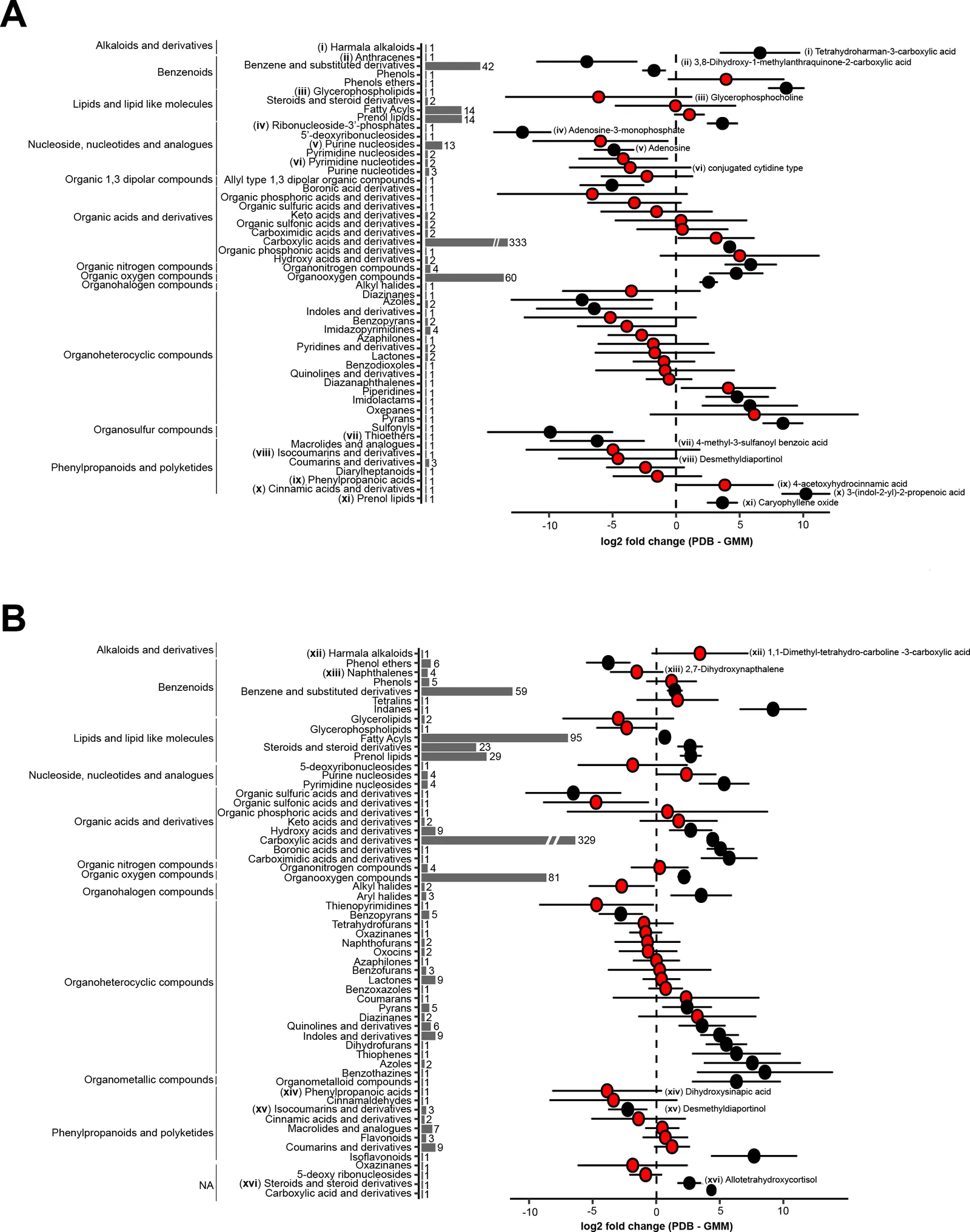
Class-level metabolite differences among nutrient media. (**A**) Aqueous extract and (**B**) organic extract of *Hyaloscypha finlandica* PMI 746 exudates. Each point represents the estimated log2 fold change between PDB and GMM for a class, derived from a mixed-effect model [value ~ group + (1 | metabolite)]. Points on the right of zero indicate higher abundance in PDB; points to the left indicate higher abundance in GMM. Error bars show 95% confidence intervals. Classes are grouped and ordered by superclass. The name of the most abundant metabolite within each class (highest mean log2 intensity across sample) is shown above its point estimate. The barplot panel shows the number of metabolites contributing to each class level model. Because one class was disproportionately large, metabolite counts were truncated, but a true count is displayed next to the bar. Red point: nonsignificant, black point: significant. All annotations are putative.

Medium composition strongly shaped the extracellular chemical footprint of *H. finlandica*, with distinct metabolite classes preferentially enriched in GMM versus PDB cultures. In the aqueous extract, GMM cultures showed higher relative abundance of several redox-active and stress-associated putative annotations, including anthracene-like features (e.g., 3,8-dihydroxy-1-methylanthraquinone-2-carboxylic acid), glycerophospholipids (e.g., glycerophosphocholine), nucleosides (e.g., adenosine), and isocoumarin-related compounds such as desmethyldiaportinol (Fig. 5A). In contrast, PDB cultures showed higher abundances of alkaloids (e.g., tetrahydroharman-3-carboxylic acid), prenol lipids (e.g., caryophyllene oxide), and phenylpropanoid-/polyketide-related features. Similar trends were observed in the organic extract (Fig. 5B). GMM cultures were enriched in redox-active naphthalenes (e.g., 2,7-dihydroxynaphthalene) and phenylpropanoid-/polyketide-related features, whereas PDB cultures showed elevated nitrogen-containing alkaloid-like features such as 1,1-dimethyl-tetrahydrocarboline-3-carboxylic acid and steroid-related annotations such as allotetrahydrocortisol. Because residual nutrients were not measured at the end of cultivation, these differences are interpreted as medium-associated metabolite responses rather than evidence of nutrient depletion or physiological nutrient limitation.

### Contextualizing *H. finlandica* metabolites using community repositories

Public metabolomics repositories enable placement of detected metabolites within broader taxonomic and ecological contexts. Using microbeMASST (68), we queried representative *H. finlandica* metabolites against publicly available data sets to assess their distribution across microbial lineages. Although limited deposition of fungal endophyte metabolomes constrains phylogenetic resolution, several informative patterns emerged. A diterpene annotated as a 7-oxoabieta-8,11,13-trien-18-oic acid feature was supported by an authentic-standard MS/MS spectral match; querying its spectrum against microbeMASST further identified matching spectra from a bacterial *Streptomyces* spp. and fungi including *Tolypocladium* sp. (Fig. S1A). Although the dominant fragment ions were shared, differences among lower-intensity ions suggest that these matches may represent structurally related diterpenoids rather than an identical compound, potentially reflecting a shared core scaffold across bacterial and fungal data sets. The putative benzopyran polyketide desmethyldiaportinol showed spectral similarity primarily to *Alternaria* data sets (Fig. S1B). In contrast, the siderophore desferricrocin exhibited closely matching signatures across diverse fungi, including *Chalciporus piperatus* in Basidiomycota and multiple genera (e.g., *Botrytis*, *Cladosporium,* and *Fusarium*) in Ascomycota, consistent with broad fungal siderophore biology (Fig. S1C).

These examples illustrate how community metabolomics resources can contextualize *H. finlandica* metabolites within the broader fungal chemical landscape. They also reinforce the need for conservative interpretation: database co-occurrence and spectral similarity can support prioritization and ecological context but require authentic standards, NMR, targeted genetics, isotope labeling, or heterologous expression for definitive structural or biosynthetic assignments.

## DISCUSSION

Dark septate endophytes are common in forest soils and influence plant growth, stress tolerance, and nutrient allocation under experimental conditions (10, 62, 69–74). Because curated fungal ecology resources are incomplete for many non-model taxa, including *Hyaloscypha*, we treated ecological assignments as working hypotheses derived from source literature, genome metadata, and nomenclature updates. Within this curated 41-genome panel, three complementary BGC prediction pipelines converged on enriched PKS-, NRPS-, and terpene-associated biosynthetic capacity in DSEs, although the strength of enrichment varied by tool and BGC class. Phylogenetically informed models were broadly consistent across taxonomy-based and genome-derived trees, with instability largely confined to sparsely represented classes.

The predictors used here captured overlapping but non-identical views of fungal biosynthetic potential. antiSMASH is conservative and anchored to MIBiG reference comparisons; FunBGCeX expands fungal detection, including domainless biosynthetic enzymes; and BGC-Prophet applies protein language modeling and yielded higher cluster counts while preserving rank concordance across genomes. Product-label agreement across tools was limited, but genome-level rank agreement was strong. This supports convergence on relative biosynthetic capacity, not on exact chemical products. Accordingly named antiSMASH or FunBGCeX labels are best interpreted as reference similarities unless supported by deeper domain analysis, synteny, and metabolite evidence.

The GCF analysis further supports the view that DSE genomes contain substantial, underexplored biosynthetic diversity. Most BiG-SCAPE families were restricted to a single genome, and only one GCF was shared across all seven primary ecological categories represented in the comparison. DSE genomes carried more NRPS GCFs and more total GCFs in rank-based tests, consistent with the BGC count enrichment, but the weaker phylogenetic support indicates that ecological guild, lineage history, and uneven sampling are difficult to separate in the current data set. Thus, the GCF results should not be interpreted as evidence for DSE-specific products; rather, they reinforce that DSE-associated biosynthetic space remains sparsely characterized and warrants broader, taxonomically balanced genome-mining paired with metabolomics.

For *H. finlandica* specifically, genome mining supported substantial PKS, NRPS, and terpene capacity. Several candidate intersections were consistent with metabolomics results. PKS/T1PKS reference similarities to DHN-melanin-related intermediates align with the biology of melanized DSEs and the detection of dihydroxynaphthalene-like features (75), but LC-MS/MS could not resolve positional isomers. The siderophore-associated NRPS candidate aligns with the detection of desferricrocin-/ferricrocin-like features and with broad fungal siderophore biology (76–78). A brefeldin-like candidate remains low confidence as reference-based genomic and spectral agreement was limited and the metabolite annotation remains to be structurally validated. The authentic-standard comparison for 7-oxodehydroabietic acid provides the strongest metabolite-level evidence. The standard-supported MS/MS match strengthens the biological relevance of terpene-associated chemistry in *H. finlandica*, particularly because comparative genome mining suggested enriched terpene capacity among DSE genomes.

Across extracts, enriched metabolite classes included peptide-like compounds, aromatic polyketides, fatty-acid-derived/lactone features, siderophore-like features, and sesquiterpenoid-like annotations. Interpreted cautiously, these patterns are consistent with (i) NRPS/hybrid activity yielding peptide-like and siderophore-type chemistries (77, 79), (ii) NR-PKS activity producing aromatic scaffolds such as benzopyrans and naphthopyrones (80–82), (iii) HR-PKS contributions to lipid-like and oxylipin-related features (83), and (iv) terpene synthases generating sesquiterpenoids, often partnered with oxidative tailoring enzymes (84). Collectively, these findings warrant targeted genetic, biochemical, and structural characterization of the secondary metabolite repertoire of *H. finlandica* and other DSEs.

These results align with prior Helotiales observations. *Sclerotinia sclerotiorum* encodes ~ 80 BGCs with infection-linked expression (85), Helotiales sp. BL73 carries extensive predicted terpene capacity (86), and *Hymenoscyphus fraxineus*/*H. albidus* show expanded repetitive content and BGC repertoires (87). Metabolomics studies likewise report peptide-like compounds, cholines, nucleobases/nucleotides, sugars, and siderophores in *Pinus sylvestris*-associated DSEs (88) and polyketide-rich chemistry in halophyte DSEs such as *Gaeumannomyces* sp. (89).

A persistent interpretive limitation in untargeted metabolomics is separating *de novo* fungal metabolites from medium-derived compounds, enzymatic transformation products, and intracellular metabolite release by leakage or partial lysis during growth and processing. Both PDB and GMM contain substrates that can be assimilated or enzymatically remodeled. The exometabolome measured here is, therefore, best interpreted as an integrated extracellular footprint of fungal growth under defined medium contexts, not as a catalog of terminal biosynthetic products (28, 90). Nonetheless, consistent enrichment relative to matched uninoculated controls and strong separation in ordination space indicate that *H. finlandica* reproducibly reshapes its extracellular chemical environment.

In summary, this analysis establishes *H. finlandica* PMI 746 as a paired genome-mining and exometabolomics reference for DSE metabologenomics. The data set supports candidate links among encoded PKS, NRPS, terpene, and siderophore capacity and observed extracellular chemical features, while explicitly distinguishing prediction, putative annotation, standard-supported metabolite evidence, and unresolved BGC-product assignment. Definitive genome-metabolite links will require targeted genetics, heterologous expression, isotope labeling, purified standards, and/or NMR. The public data sets and code are intended to support community-driven exploration of DSE specialized metabolism without over-assigning function in a context-dependent fungal guild.

## Supplementary Material

Reviewer comments

## Data Availability

Raw LC-MS/MS data are available through the MassIVE repository under accession number MSV000099765, and the Paired Omics Data Platform (PODP) data are recorded at https://pairedomicsdata.bioinformatics.nl/projects/deffa5a6-9d05-4b8f-b12a-fc8fa7a57e76.2. Processed feature tables, annotation tables with confidence levels, genome-mining summaries, GCF outputs, and analysis scripts are available at https://doi.org/10.25983/PMI/3366812.

## SUPPLEMENTAL MATERIAL

Material S1 msystems.00838-26-s0001.docx Metabolomics workflow.

Material S2 msystems.00838-26-s0002.xlsx Fungal data set validation and search parameters.

Figure S1 msystems.00838-26-s0003.docx microbeMASST search of top compounds.

Figure S2 msystems.00838-26-s0004.docx Chemical standard validation.

Table S1 msystems.00838-26-s0005.xlsx ClassyFire annotations.

OPEN PEER REVIEW reviewer-comments.pdf An accounting of the reviewer comments and feedback.

ASM does not own the copyrights to Supplemental Material that may be linked to, or accessed through, an article. The authors have granted ASM a non-exclusive, world-wide license to publish the Supplemental Material files. Please contact the corresponding author directly for reuse.
