# Supplementary material for: Genome mining and metabolomics reveal context-dependent biosynthetic potential in a core poplar dark septate endophyte
Source: mSystems. 2026 Sep 23;11(10):e00838-26. doi: 10.1128/msystems.00838-26 (PMC13712853; doi:10.1128/msystems.00838-26)
Supplement: Supplementary file 1 [file reviewer-comments.pdf]

ASM | mSystems

Genome mining and metabolomics reveal context-dependent biosynthetic potential in a core poplar dark septate endophyte

Julian Cosner, Sameer Mudbhari, Jack Orebaugh, Robert Hettich, Paul Abraham, and Tomas Rush

Corresponding Author(s): Tomas Rush, Oak Ridge National Laboratory

Review Timeline:	Submission Date:	June 30, 2026
	Editorial Decision:	August 2, 2026
	Revision Received:	August 20, 2026
	Accepted:	August 21, 2026

Editor: Justin van der Hooft

Reviewer(s): Disclosure of reviewer identity is with reference to reviewer comments included in decision letter(s). The following individuals involved in review of your submission have agreed to reveal their identity: Esteban Charria Giron (Reviewer #2)

Transaction Report:

(Note: With the exception of the correction of typographical or spelling errors that could be a source of ambiguity, letters and reports are not edited. The original formatting of letters and referee reports may not be reflected in this compilation.)

DOI: https://doi.org/10.1128/msystems.00838-26

Re: mSystems00838-26 (Genome mining and metabolomics reveal context-dependent biosynthetic potential in a core poplar dark septate endophyte)

Dear Dr. Tomas Allen Rush:

Thank you for the privilege of reviewing your work. Below you will find my comments, instructions from the mSystems editorial office, and the reviewer comments.

The revision answers most of the criticism of the reviewers. Please follow the remaining suggestions before the manuscript can be accepted for publications.

Please return the manuscript within 60 days; if you cannot complete the modification within this time period, please contact me. If you do not wish to modify the manuscript and prefer to submit it to another journal, notify me immediately so that the manuscript may be formally withdrawn from consideration by mSystems.

Revision Guidelines
To submit your modified manuscript, log into the submission site at https://msystems.msubmit.net/cgi-bin/main.plex. Go to Author Tasks and click the appropriate manuscript title to begin. The information you entered when you first submitted the paper will be displayed; update this as necessary. Note the following requirements:

• Upload point-by-point responses to the issues raised by the reviewers in a file named "Response to Reviewers," NOT in your cover letter.
• Upload a compare copy of the manuscript (without figures) as a "Marked-Up Manuscript" file.
• Upload a clean .DOC/.DOCX version of the revised manuscript and remove the previous version.
• Each figure must be uploaded as a separate, editable, high-resolution file (TIFF or EPS preferred), and any multipanel figures must be assembled into one file.
• Any supplemental material intended for posting by ASM should be uploaded with their legends separate from the main manuscript. You can combine all supplemental material into one file (preferred) or split it into a maximum of 10 files with all associated legends included.

For complete guidelines on revision requirements, see our Submission and Review Process webpage. Submission of a paper that does not conform to guidelines may delay acceptance of your manuscript.

Data availability: ASM policy requires that data be available to the public upon online posting of the article, so please verify all links to sequence records, if present, and make sure that each number retrieves the full record of the data. If a new accession number is not linked or a link is broken, provide mSystems production staff with the correct URL for the record. If the accession numbers for new data are not publicly accessible before the expected online posting of the article, publication may be delayed; please contact production staff (mSystems@asmusa.org) immediately with the expected release date.

Publication Fees: For information on publication fees and which article types are subject to charges, visit our website. If your manuscript is accepted for publication and any fees apply, you will be contacted separately about payment during the production process; please follow the instructions in that e-mail. Arrangements for payment must be made before your article is published.

ASM Membership: Corresponding authors may join or renew ASM membership to obtain discounts on publication fees. Need to upgrade your membership level? Please contact Customer Service at Service@asmusa.org.

The ASM Journals program strives for constant improvement in our submission and publication process. Please tell us how we can improve your experience by taking this quick Author Survey.

Thank you for submitting your paper to mSystems.

Sincerely,
Justin van der Hooft
Editor
mSystems

Reviewer #2 (Comments for the Author):

Dear Authors,

Thank you for your efforts and for considering most of our comments. I believe the current manuscript has improved significantly

compared to the previous version. It now properly acknowledges the data depth of each individual omic and therefore does not overstate intermediate findings beyond what the data actually support. Overall, the present study higlights the need for future work on DSEs and makes clear that studying biosynthetic potential and chemical diversity in this group of fungi deserves particular emphasis.

I have added minor comments to the attached document, which can help polish some passages and make some statements a bit more solid, but otherwise I believe the study is a solid contribution to the field and can serve as a nice reference point for future studies.

Please make sure all links are openly accesible, such as the GitHub repository, which I could not access, but besides this, it is very nice to see your efforts towards and fair and open data.

Title: Genome mining and metabolomics reveal context-dependent biosynthetic potential in a core poplar dark septate endophyte

Target Journal: mSystems

Authors: Julian B. Cosner[1ⴕ], Sameer Mudbhari[1,2,ⴕ], Jack A. Orebaugh[1,2], Robert L. Hettich[1], Paul E. Abraham[1,*], Tomás A. Rush[1,*]

[1]Biosciences Division, Oak Ridge National Laboratory, Oak Ridge, TN 37831, USA
[2]Bredesen Center for Interdisciplinary Research and Graduate Education, University of Tennessee, Knoxville, Tennessee, USA

ⴕauthors contributed equally
[*]Corresponding authors - abrahampe@ornl.gov; rushta@ornl.gov

Notice: This manuscript has been authored by UT-Battelle, LLC, under contract DE-AC0500OR22725 with the US Department of Energy (DOE). The US government retains and the publisher, by accepting the article for publication, acknowledges that the US government retains a nonexclusive, paid-up, irrevocable, worldwide license to publish or reproduce the published form of this manuscript, or allow others to do so, for US government purposes. DOE will provide public access to these results of federally sponsored research in accordance with the DOE Public Access Plan (http://energy.gov/downloads/doe-public-access-plan).

Main Text word count: 5,341

Abstract word count: 235

Keywords: dark septate endophytes, poplar, fungal metabolome, secondary metabolite, biosynthetic gene cluster, plant-microbe interactions

Abstract

Dark septate endophytes (DSEs) are ubiquitous root-colonizing fungi characterized by melanized, septate hyphae and a frequent associations with plants in stressful or nutrient-poor environments. Although DSEs are increasingly recognized for context-dependent effects on host stress resilience, the specialized metabolic capacity of many DSE lineages remains poorly resolved. *Hyaloscypha finlandica* is a recurrent root-associated fungus of *Populus* and other hosts, making it a useful system for understanding symbiotic mechanisms using multi-omics analyses. Here, we combined comparative biosynthetic gene cluster (BGC) prediction across 41 ascomycete genomes with untargeted metabolomics of *H. finlandica* PMI 746 grown in a defined minimal medium and a complex plant-derived medium. Benchmarking antiSMASH, FunBGCeX, and BGC-Prophet revealed broad conservation of PKS-, NRPS-, and terpene-associated biosynthetic capacity among DSEs, while also showing that tool-specific reference-product labels vary and should be interpreted conservatively. LC-MS/MS profiling detected strong medium-associated shifts in the extracellular chemical footprint of *H. finlandica* and identified putative terpenoid-, polyketide-, peptide-, siderophore-, and DHN-melanin-related features through spectral matching and *in silico* chemical-class inference. Authentic-standard comparison supported a probable assignment of 7-oxodehydroabietic acid, whereas most other annotations remain putative or class-level assignments. Integrating genomic and metabolomic evidence identified representative candidate intersections between encoded biosynthetic capacity and observed chemistry, but definitive BGC-product links remain targets for future genetic and structural validation. Together, these results provide a public paired-omics resource for a core *Populus*-associated DSE and a conservative framework for interpreting fungal multi-omics in non-model endophytes.

IMPORTANCE. DSEs are widespread fungal symbionts that influence plant performance, yet their encoded and expressed metabolic capacities remain largely uncharacterized. This study provides a paired comparative genome-mining and untargeted metabolomics resource for *Hyaloscypha finlandica*, a recurrent *Populus*-associated DSE. By explicitly distinguishing BGC prediction, spectral annotation, authentic-standard support, and unresolved BGC-prophet assignment, the work provides a transparent reference for future studies of DSE specialized metabolism. The resulting datasets support comparative analyses across fungal endophytes and help prioritize candidate metabolites and gene clusters for targeted validation.

1 Introduction

Root-associated fungi are central to plant nutrition, stress tolerance, and ecosystem resilience. Beyond well-studied mycorrhizal symbionts, class IV endophytes, commonly referred to as dark septate endophytes (DSEs), are widespread but comparatively understudied root-associated fungi defined by melanized, septate hyphae and intraradical microsclerotia that occur across diverse plant hosts and ecosystems, particularly under stressful or nutrient-poor conditions (1-8). Despite their ubiquity, the mechanisms and chemical mediators underlying DSE-plant interactions remain poorly resolved. DSEs are frequently isolated from woody and herbaceous plants worldwide (9-11). Their recurrence across woody and herbaceous hosts, including communities dominated by ectomycorrhizal and arbuscular mycorrhizal fungi, indicates that DSEs represent a distinct component of plant root microbiomes (12-14).

Among DSEs, *Hyaloscypha finlandica* [(C.J.K. Wang & H.E. Wilcox) Vohnik, Fehrer & Réblová], (syn: *Phialophora finlandia, Cadophora finlandica*), has emerged as a broadly distributed and functionally relevant species (15-17). Although many *Hyaloscypha* species are described as saprobes of wood and leaf litter, *H. finlandica* frequently colonizes roots of ericoid, hardwood, and conifer hosts, where it can form mycorrhizal-like and endophytic associations (15, 17-24). Depending on strain, host, and growth context, *H. finlandica* can have positive (10, 23, 25, 26) or negative (27) effects on plant growth. Recent time-series metabarcoding analyses suggest that *H. finlandica* is a recurrent and active root-associated fungus of *Populus tremuloides* across geographical gradients (14), further underscoring its ecological relevance as a DSE for multi-omics. Yet key biological mechanisms, including host specificity, colonization, metabolic activity, and nutrient exchange, remain unresolved for this and other DSE species (1, 3, 9).

Secondary metabolites (SMs) mediate competition, signaling, nutrient acquisition, and stress responses in plant-fungal interactions (28-31). Genome-scale mining enables automated detection and comparison of biosynthetic gene clusters (BGCs), offering a first-pass view of encoded biosynthetic potential (32). However, BGC prediction is not equivalent to production or product assignment. Most predicted fungal BGCs lack experimentally characterized products: in a large fungal genome atlas, only a small fraction of gene-cluster families could be linked to known metabolites (33), and machine-learning models remain constrained by limited validated training data despite estimates that Pezizomycotina alone may encode millions of secondary metabolites (34, 35).

Untargeted LC-MS/MS metabolomics provides a complementary view of expressed chemical output (36-39), but metabolite annotation is limited by incomplete spectral libraries, adduct/isomer ambiguity, and the inability of MS/MS alone to resolve many stereochemical and positional variants. As a result, genomics often predicts metabolites that remain chemically unobserved, while metabolomics detects compounds lacking genomic attribution. Recent advances in fragmentation modeling, molecular fingerprint prediction, and neural network-based classification, particularly within the SIRIUS framework, have expanded annotation coverage by enabling inference of molecular formulas, substructures, and chemical classes (40, 41). Complementary tools such as GNPS molecular networking and NPLinker help prioritize genome-metabolite links, but candidate links still require targeted validation (42-46).

Few studies have profiled metabolites directly from cultured DSE fungi, with most focusing instead on host responses or plant-facing chemistry (47-50). While some DSE taxa, such as *Periconia* spp., have genomic resources (51, 52), paired genome-mining and metabolomics datasets remain rare.

Motivated by the scarcity of DSE metabolomic references and the need to connect encoded biosynthetic potential with expressed chemistry, we addressed two questions: (i) do DSEs and closely related taxa exhibit consistent genomic capacity for secondary metabolism across lineages and literature-supported ecological categories, and (ii) does the extractable exometabolome of *H. finlandica* respond to defined culture media in ways consistent with this biosynthetic capacity? To answer these questions, we combined comparative genome mining across DSE and related ascomycetes with untargeted LC-MS/MS exometabolomics of *H. finlandica* PMI 746. We present this dataset as a public paired-omics resource and as a conservative framework for prioritizing candidate BGC-metabolite relationships in non-model fungal endophytes.

2 Materials and Methods

2.1 Fungal culture and growth conditions

Hyaloscypha finlandica strain PMI 746, originally isolated as a root endophyte from *Populus deltoides* (53), was used in all experiments. This strain was maintained as a 50% glycerol stock at -80°C and then subcultured on potato dextrose agar (PDA; BD Difco, New Jersey) prior to use. For initial plate cultures, 1.0 cm^2 mycelial fungal plugs were aseptically excised from the colony edge using a flame-sterilized cork borer and used to inoculate 10 new plates (100 x 15 mm) of PDA and 10 plates of glucose minimal medium (GMM) lacking thiamine (54). Plates were incubated at 25°C in the dark for 10 days. The resulting biomass was used for elemental profiling in Rush et al. (55). For liquid culture, fresh 1.0 cm^2 plugs from *H. finlandica* were excised from each respective media and individually inoculated into 125 mL Erlenmeyer screw-cap flasks containing filled 50 mL of either potato dextrose broth (PDB; BD Difco, New Jersey) or GMM. Cultures were incubated in the dark at 25°C for 21 days at 250 rpm in an Eppendorf New Brunswick Excella E25 shaking incubator. PDB was used as a complex, plant-derived rich

medium, whereas GMM was used as a defined minimal medium. For each medium, five biological replicates and five medium-only controls (no agar cores) were prepared. After incubation, cultures were vacuum-filtered through 0.45 µm membranes into sterile 50 mL Falcon tubes using a Millipore® Steriflip® vacuum system (SE1M003M00). Filtrates were stored at -80°C overnight, then freeze-dried (Labconco™ FreeZone™ 2.5 L Benchtop Freeze Dryer) with ventilated lids until completely dried.

2.2 Metabolite extraction

Lyophilized fungal spent media were reconstituted in a 1:1 (v/v) mixture of HPLC-MS grade water and ethyl acetate (1 mL each), vortexed, and phase-separated into two sterile vials. The aqueous extract was re-frozen and dried by lyophilization, while the ethyl acetate fraction was air-dried under sterile conditions. Dried aqueous extracts were resuspended in HPLC solvent A (98% water, 2% acetonitrile, 0.1% formic acid), and dried ethyl acetate extracts were resuspended in HPLC solvent B (70% acetonitrile, 30% water, 0.1% formic acid).

2.3 Metabolite measurements and standard comparisons

Metabolomics data were acquired by liquid chromatography tandem mass spectrometry (LC-MS/MS) on a Vanquish ultra-high performance liquid chromatography (UHPLC) system (Thermo Fisher Scientific) coupled to a Q Exactive Plus mass spectrometer (Thermo Fisher Scientific) following procedures described previously (39). In brief, chromatographic separations were performed using a 75 µm inner diameter glass capillary packed in-house to 15 cm with Kinetex C18 beads (1.7 µm particle size, 100 Å pore size), using a 30 min gradient from aqueous to organic solvent (Solvent B increasing from 0% to 100% over 0-15 min, held at 100% from 15-16 min, then decreased to 0% from 16-18 min and held at 0% until 30 min). Mass spectra were acquired in positive ionization mode with full MS resolution of 70,000 and MS/MS resolution of

17,500 over an m/z range of 135-2000. A data-dependent acquisition (DDA) method was used in which the top five most intense precursor ions per MS scan were selected for fragmentation, with dynamic exclusion of 10 s and stepped normalized collision energies of 10, 20, and 40 eV. Data were acquired using XCalibur v4.3 (Thermo Fisher Scientific). Full instrument setup and parameters are provided in **Supplemental Material S1**.

Commercially available standards were purchased and measured for two prioritized metabolites: 1,3,6,8-tetrahydroxynaphthalene and 7-oxodehydroabietic acid. Standards were analyzed under the same LC-MS/MS platform and compared with *H. finlandica* features using precursor mass, retention behavior where available, and MS/MS mirror plots (**Supplemental Fig. S2**). The 7-oxodehydroabietic acid standard produced a confident standard-supported MS/MS match to the corresponding *H. finlandica* feature. The 1,3,6,8-tetrahydroxynaphthalene comparison is reported in the supplement, but positional/isomeric ambiguity was not fully resolved by LC-MS/MS alone. We therefore refer to 7-oxodehydroabietic acid as a standard-supported probable annotation and retain conservative language for other features unless orthogonal structural validation is available.

2.4 Metabolite data processing and annotation confidence

Raw LC-MS/MS files were processed in Compound Discoverer v3.3 (Thermo Fisher Scientific). Aqueous and organic extracts were analyzed separately because solvent-specific retention behavior prevents reliable cross-fraction feature alignment. For each fraction, 20 raw files (five biological replicates and five uninoculated controls per medium) were processed independently. The Compound Discoverer workflow included spectral selection, peak detection, feature grouping, compound annotation, and chemical composition prediction; full parameter settings are provided in **Supplemental Material S1**. In parallel, molecular networking and in silico

annotation were performed using GNPS2 (56) and SIRIUS v6.3 (57). Raw files were converted to .mzML format using MSConvert v3.0 with vendor peak picking (MS1-MS2). Classical molecular networking was run separately for aqueous and organic extracts in GNPS2 to generate consensus MS/MS spectra (.mgf), which were then used as input for SIRIUS.

SIRIUS outputs, including molecular formulae and CANOPUS-derived compound class predictions, were merged with the Compound Discoverer feature tables using rounded m/z values (three decimal places) and ion mass information. The structure_identification.tsv file was integrated in R v4.5.1 using a fuzzy join with an m/z tolerance of 0.001 Da to account for minor mass reporting differences. For features annotated by spectral matching to the NIST 2023 and GNPS libraries, chemical classification was assigned using InChIKeys for the closest retrievable compounds in PubChem or ChemSpider, followed by batch classification with ClassyFire (58). Spectral matches were retained only when (i) mass deviation was consistent with common adducts or analogs within ±60 Da, and (ii) the NIST HighChem HighRes score was ≥70 and represented the top-ranked match for that feature. Annotation confidence was assigned using metabolomics reporting conventions derived from the Metabolomics Standards Initiative and subsequent small-molecule annotation frameworks (59, 60). Features supported by authentic-standard LC-MS/MS comparison are reported as standard-supported probable annotations; library matches without standards are reported as putative annotations; SIRIUS/CANOPUS and ClassyFire outputs are reported as formula, substructure, or class-level annotations; and unannotated features are retained as molecular features. We avoid treating library or in silico annotations as confirmed structures.

2.5 Metabolomics statistical analysis

Untargeted metabolomics data from *H. finlandica* aqueous and ethyl acetate extracts were processed separately using a standardized statistical workflow in R v4.5.1 with tidyverse, vegan, rstatix, ggpubr, ggfortify, and reshape2. Peak area matrices and metadata exported from Compound Discoverer were condensed using an in-house script (https://github.com/dlcarper/Metabolite-Condensing-Script) to group features within a 0.05 Da m/z tolerance. Zero values were converted to NA, features containing only missing values were removed, and intensities were Log2-transformed. Signal normalization was performed in two stages. First, LOESS normalization was applied using replicate metadata to correct sample-specific drift; residuals from the fitted models were retained as normalized intensities. Second, features were median centered across samples to remove remaining global offsets from differences in biomass production and injection load. Sample-wise intensity distributions were inspected to confirm comparable signal ranges across runs. Missing values were imputed under a left-censored, missing-not-at-random assumption using a downshifted Gaussian distribution (width = 0.3, downshift = 2.4; seed = 100).

Multivariate structure was assessed using Bray-Curtis dissimilarities calculated from the normalized, imputed feature matrix. Non-metric multidimensional scaling (NMDS) was performed with the metaMDS algorithm using multiple random starts to ensure convergence. Ordination scores were merged with sample metadata and visualized using group-specific colors and shapes with 95% confidence ellipses. For univariate analysis, each feature was modeled independently as a function of experimental group using normalized, imputed Log2 intensities. Linear models of the form intensity ~ group were fit, and one-way ANOVA was used to test for overall group effects. P-values were adjusted using the Benjamini-Hochberg procedure; features with FDR-adjusted $p < 0.05$ were considered significant. Tukey HSD post hoc tests were applied

to significant features to resolve pairwise contrasts. For effect size interpretation, Log2 intensities were back-transformed to approximate raw-scale abundances, and fold changes were calculated as ratios of group means.

Class-level differences between PDB and GMM conditions were quantified using mixed-effects models. Processed Log2-transformed intensities were imported from a curated dataset, and metabolite-specific abundances were modeled as value ~ Group + (1 | Metabolite). Classes represented by a single metabolite were analyzed using fixed-effects models. Estimated marginal means and contrasts (PDB - GMM) were computed with emmeans. Log2 fold-change estimates, 95% confidence intervals, and p-values were adjusted for multiple testing using Benjamini-Hochberg correction. For visualization, each class was annotated with the number of contributing metabolites, and the most abundant metabolite per class (highest mean Log2 intensity) was labeled at the corresponding point estimate in forest plots.

2.6 Fungal genome dataset acquisition

A comparative genome-mining approach was used to assess secondary metabolic potential across DSE fungi and related taxa and to identify conserved BGC features. Based on published surveys of DSE diversity (3, 51, 61-63), we curated a dataset representing described DSE species and closely related ascomycetes, including melanized and non-melanized outgroups spanning diverse ecological lifestyles (e.g., mycorrhizal symbionts, endophytes, saprotrophs, and opportunistic pathogens). Ecological categories were literature-derived, strain/source-associated annotations based on primary literature and genome metadata, not fixed organismal identities. These categories were used for exploratory comparisons, recognizing that fungal lifestyles can be context-dependent and may shift with host, substrate, developmental stage, and environment.

A total of 41 ascomycete genomes were retrieved from NCBI GenBank, including 19 DSE genomes and 22 ecological outgroups. For each assembly, the genomic FASTA, annotation (.gff), and predicted protein sequences (.faa) were downloaded using the NCBI Datasets command-line tool. Species names were validated against Index Fungorum (*indexfungorum*.org) and Mycobank (*mycobank*.org), and genome accessions with ecological annotations and supporting sources are provided in **Supplemental Material S2**.

2.7 Mining biosynthetic potential

BGCs were identified using three complementary genome-mining pipelines representing distinct methodological paradigms: antiSMASH (rule-based; 64), FunBGCeX (profile/HMM-based; 65), and BGC-Prophet (deep learning-based; 66). All tools were run with default or recommended parameters. Tool outputs were interpreted as predicted biosynthetic capacity and reference-cluster similarity, not direct product identification.

antiSMASH v8.0.1 was executed via the fungal web server with detection strictness set to relaxed to retain partial or atypical clusters (e.g., NRPS-like). For each genome, antiSMASH reported predicted BGC regions with genomic coordinates, cluster class assignments (e.g., PKS, NRPS, RiPP, terpene), hybrid designations, and core biosynthetic genes. KnownClusterBlast was used to compare predicted clusters to the MIBiG v4.0 database, and the most similar reference cluster and confidence level were recorded as reference similarities. Cluster counts and annotations were extracted from GenBank outputs.

FunBGCeX v1.0.0 was run following the authors' published workflow. The pipeline identifies biosynthetic genes of interest, including canonical core enzymes and domainless biosynthetic proteins, and evaluates flanking genomic regions for additional biosynthetic signatures using an HMMER v3.4 library and DIAMOND v2.2.0 searches against a curated BGC

protein database. Regions meeting predefined distance and gene-content criteria and co-localized with core biosynthetic genes were reported as BGCs.

BGC-Prophet v0.1.0 was installed in a Conda environment and ran in full mode using pre-trained detection and classification models (annotator.pt and classifier.pt). Predicted protein sequences (.faa) were used as input. The pipeline embeds protein sequences using the ESM2 language model, predicts BGC-associated genes at a probability threshold of 0.5, merges adjacent predictions into clusters (maximum gap of three genes; minimum cluster size of two genes), and assigns product classes. Output CSV files reported predicted BGCs with confidence scores and annotations.

2.8 Genome mining analysis

BGC predictions from all tools were parsed and compiled into tool-specific tables containing a unique BGC identifier, species name, ecological category, region designation (for antiSMASH), cluster class, genomic coordinates, and associated reference-product information. Genome and proteome assemblies were evaluated using BUSCO v6.0.0 with the ascomycota_odb12 lineage dataset (2,826 single-copy orthologs). Only genomes with ≥70% complete BUSCO scores were retained. A taxonomic reference tree was generated using the NCBITaxa ete3 v3.1.3 function based on NCBI TaxIDs for all 41 fungi. Hierarchical relationships were retrieved, strain-level leaves were duplicated as needed, and the resulting topology exported in Newick format (.nwk) for visualization in iTOL v7.2.2.

BGC predictions were aggregated across genomes to compare per-genome cluster counts and class composition by tool and ecological category. Statistical comparisons among ecological groups were conducted using ANOVA with pairwise post hoc tests, alongside non-parametric Wilcoxon tests with Benjamini-Hochberg correction within tool. Phylogenetically informed

regressions were performed using the phylolm v2.6.5 package in R under a lambda model, equivalent to PGLS when the same lambda is applied. Analyses were repeated using both the NCBI taxonomy-based tree and a genome-derived MashTree v1.4.6 phylogeny (67) to assess sensitivity to tree choice.

For antiSMASH outputs, GenBank files were parsed with Biopython to extract cluster classes and genomic coordinates. Custom Python scripts assigned clusters to canonicalized classes (NRPS, PKS, RiPP, terpene, indole, saccharide, alkaloid, hybrid, other, unknown), retaining original annotations for traceability. Regions with multiple or class-like assignments were classified as hybrids. Genome sizes were calculated from assembled FASTA files and included as reference metadata. All genome-mining data processing and statistical analyses were performed using custom Python v3.12.3 and R v4.4.2 scripts, with visualization in ggplot2. To avoid equating raw BGC counts with biosynthetic diversity, predicted clusters from antiSMASH were additionally grouped into gene cluster families (GCFs) using sequence-similarity and shared-biosynthetic-gene context across predicted regions using BiG-SCAPE v2.0.3. As this genome-mining approach was exploratory rather than confirmatory, candidate *H. finlandica* clusters corresponding to prioritized metabolite annotations were not inspected for domain architecture or synteny relative to MIBiG or literature reference clusters. Because fungal clusters with similar domain architecture can produce distinct products, BGC totals and GCF membership were used to assess relatedness and candidate plausibility rather than to claim product identity.

3 Results

3.1 Comparative biosynthetic potential among DSEs and related ascomycetes

Using antiSMASH, FunBGCeX, and BGC-Prophet, we observed tool-specific differences in both the number and class composition of predicted BGCs, yet strong rank concordance across 41 sampled ascomycete genomes (**Fig. 1A**). Across the datasets, the three approaches predicted 1,715 (antiSMASH), 1,886 (FunBGCeX), and 4,524 (BGC-Prophet) total BGCs, corresponding to means of 41.8, 46.0, and 110.3 clusters per genome, respectively. Genome-level totals were highly correlated between antiSMASH and FunBGCeX (Spearman $\rho = 0.964$, $p < 4e\text{-}24$) and moderately correlated between each of those tools and BGC-Prophet ($\rho = 0.645$, $p < 5e\text{-}6$; $\rho = 0.573$, $p < 9e\text{-}5$), respectively. Genome size was positively associated with BGC counts for all tools: antiSMASH ($\rho = 0.60$, $p < 3e\text{-}5$); FunBGCeX ($\rho = 0.54$, $p < 2e\text{-}4$); and BGC-Prophet ($\rho = 0.82$, $p < 4e\text{-}11$). These results indicate that larger genomes generally encode more predicted clusters. Class composition was method dependent: antiSMASH totals were enriched for terpenes (30.4%), PKS (29.2%), and NRPS (26.1%); FunBGCeX for PKS (28.5%), NRPS (23.9%), and terpenes (19.2%); and BGC-Prophet for PKS (29.4%), terpenes (17.3%), Unknown (16.5%), Other (12.5%), and Hybrid (11.6%) (**Fig. 1B**).

Leotiomycetes and Dothideomycetes had the highest mean per-genome BGC counts (**Fig. 1C**). Literature-derived ecological grouping showed similar trends: DSE genomes harbored higher NRPS and terpene cluster counts for several tools, whereas differences in PKS and fungal RiPP counts were inconsistent and often tool dependent (**Fig. 1D**; **Fig. 2**).

3.2 Enrichment of terpene-, PKS-, and NRPS-associated BGCs in DSE genomes

After Benjamini-Hochberg adjustment, DSE genomes had significantly more NRPS counts in antiSMASH ($q = 0.0093$; means: 13.5 vs 8.7), while the terpene difference in antiSMASH was

not significant ($q = 0.075$) (**Fig. 2**). In FunBGCeX, NRPS ($q = 0.0018$) and terpene ($q = 0.0070$) counts were higher in DSEs; PKS, RiPP, and hybrid counts were not significant after correction. BGC-Prophet predicted enrichment for PKS ($q = 0.0188$) and terpenes ($q = 0.0188$) in DSEs (means: PKS 38.4 vs 27.3; terpenes 22.8 vs 15.8). To account for shared ancestry, we used phylogenetic generalized least-squares (PGLS) models with both taxonomy-based and genome-derived trees. Model convergence (77%) was achieved for all major BGC class x tool combinations. DSE enrichment persisted for NRPS and terpenes in the genome-derived models (FunBGCeX NRPS p = 0.0099; FunBGCeX terpenes p = 0.0071; antiSMASH terpenes p = 0.038), and the taxonomy tree similarly supported NRPS enrichment (FunBGCeX NRPS p = 0.0469) while also identifying more RiPPs detected in DSEs (FunBGCeX RiPP p = 0.0264).

At the gene-cluster family level, BiG-SCAPE grouped 1,715 antiSMASH regions into 1,245 GCFs, most of which were single-genome singletons (n = 989). DSE genomes had higher total GCF counts than other genomes ($q = 0.0196$; means: 50.2 vs 34.5), including higher NRPS GCF counts ($q = 0.0196$; means: 13.9 vs 9.3). These family-level differences were not significant after phylogenetic correction (NRPS PGLS p = 0.248 with the taxonomy tree and p = 0.0895 with the genome-derived tree), indicating that the GCF signal supports the BGC count patterns but remains partly confounded with phylogenetic structure.

3.3 Reference-product labels differ among genome-mining tools

Predicted reference-product labels differed widely between antiSMASH (n = 115) and FunBGCeX (n = 286) with relatively few exact annotation overlaps (n = 31). Even among shared labels, genome-level agreement was modest (Jaccard index = 0.41; frequency-weighted = 0.46), with only six compounds showing identical co-prediction. These patterns indicate that the tools converge more strongly on relative biosynthetic capacity and broad BGC class than on specific

reference-product labels. We therefore treat named labels such as squalestatin, resorcylic acid, arthripenoids, clavaric acid, triacetylfusarinine and 1,3,6,8-tetrahydroxynaphthalene as reference similarities from genome-mining databases, not as direct evidence that the same compounds are produced by the queried fungi (**Fig. 3**). Few shared labels (e.g., abscisic acid, YWA1, choline) were observed and were primarily associated with conserved cluster families rather than direct tool-specific product agreement.

3.4 Predicted metabolic repertoire of *H. finlandica*

All three tools converged on a large and diverse predicted BGC repertoire for *H. finlandica* (**Fig. 1E**), The genome encoded multiple NRPS, PKS, and terpene-class clusters, with BGC-Prophet contributing substantial numbers of Unknown and Hybrid designations. antiSMASH generated several high-confidence reference similarities (e.g., clavaric acid-, YWA1-, PR-toxin-, and 1,3,6,8-tetrahydroxynaphthalene-like regions), whereas FunBGCeX produced a wider array of reference similarities including arthripenoid-, YWA1-, ascochlorin-, atranorin-, and abscisic acid-like regions. Notably, FunBGCeX reported both a cosmosporaside C-like transferase region found exclusively in Helotiales DSEs within the dataset and a hydroxyferricrocin-like NRPS region in *H. finlandica* with confident reference similarities (score>70).

3.5 Exometabolomic profiling of *H. finlandica* spent media

Untargeted metabolomics of *H. finlandica* spent media detected 33,777 aqueous and 26,897 organic features with quantifiable signal across cultures and media controls. These features, defined as ions observed at least once across all samples, formed the basis for downstream statistical and annotation analyses. Nonmetric multidimensional scaling (NMDS) revealed clear separation among GMM, PDB, and their respective media controls in both fractions (**Fig. 4A**), indicating strong effects of nutrient context and fungal activity on metabolite profiles.

Feature-wise one-way ANOVA with Benjamini-Hochberg correction identified 9,197 aqueous (27.2%) and 5,238 organic (19.5%) features with significant group effects (FDR < 0.05). These signals likely represent a mixture of fungal-derived metabolites, fungal-modified media constituents, lysed cell products, and other condition-dependent compounds. To enrich for fungal-associated metabolites, cultures were compared directly to matched media blanks (**Fig. 4B**). In the aqueous extract, 1,351 features were enriched in GMM-derived exudates and 1,578 in PDB-derived exudates relative to their respective blanks (Tukey HSD < 0.05, Log2 fold change ≥ 2). In the organic extract, 1,248 features were enriched in GMM cultures and 2,106 in PDB cultures. Across both fractions, this yielded 2,705 nonredundant aqueous and 2,638 nonredundant organic features enriched in at least one fungal-versus-blank contrast, representing strong candidates for *H. finlandica*-associated extracellular features.

Structural annotation was achieved through a combination of spectral library matching and in silico prediction. High-confidence spectral matches (HighChem HighRes ≥70, ±60 Da analog/adduct allowance) yielded putative identities for 5.9% of aqueous and 9.2% of organic features using NIST 2023 and GNPS libraries. To extend coverage, SIRIUS-based predictions enabled 22.2% of aqueous and 33.4% of organic features to be assigned to at least the ClassyFire superclass level (**Fig. 4C**). These values reflect annotation coverage, not confirmed structural identification.

Chemical classification revealed a broad and diverse metabolite repertoire (**Supplemental Table S1**). Carboxylic acids and derivatives dominated the annotated space, driven largely by amino acids, peptides, and related analogs. Fatty acyls and other primary metabolism-associated classes were also abundant. Multiple features corresponded to secondary metabolite classes or precursors, including linoleic acid derivatives and sesquiterpenoid-like annotations, consistent

with genome-predicted biosynthetic capacity for PKS-, NRPS-, and terpene-associated biosynthesis.

3.6 Authentic-standard and representative metabolite annotation support

Authentic-standard comparisons strengthened annotation confidence for one prioritized metabolite. The 7-oxodehydrobietic acid standard produced a close MS/MS mirror match to the *H. finlandica* feature and is therefore reported as a standard-supported probable annotation (**Supplemental Fig. S2**). Because no single syntenic terpene BGC could be assigned to this feature, it is not treated as a resolved BGC product. The 1,3,6,8-tetrahydroxynaphthalene standard comparison is also reported in the supplement, but the observed *H. finlandica* dihydroxynaphthalene-like feature could not be resolved to a unique positional isomer using LC-MS/MS alone. We therefore describe DHN-melanin-related features conservatively as putative aromatic intermediates. Similarily, library-based annotations such as desmethyldiaportinol, brefeldin C-like features, desferricrocin/ferricrocin-like features, glycerophosphocholine, resorcinol-O, O-diacetic acid-like features, and caryophyllene oxide remain putative without independent support by standard or orthogonal structural evidence.

3.7 Integrating genome-mining predictions with metabolite annotations

Direct alignment between genome-mining predictions and untargeted metabolomics remains challenging because the two approaches define chemical similarity using fundamentally different criteria. Genome-mining tools report reference similarities based on biosynthetic homology, core-gene class, domain architecture, and local gene content, whereas metabolomics annotations rely on precursor mass, fragmentation similarity, substructure inference, and library-specific nomenclature. As a result, predicted BGC product labels and detected metabolites often lack shared naming vocabularies, even when they derive from related chemical scaffolds.

To address this mismatch, we harmonized predicted BGC product labels with LC-MS/MS annotations using a canonicalization and manual curation workflow that normalized compound names, collapsed synonyms, and incorporated substructure-level classifications. This approach revealed several representative intersections between encoded capacity and detected features, including DHN-melanin-related intermediates consistent with PKS/T1PKS predictions, siderophore-associated NRPS products matching ferricrocin/desferricrocin-like annotations, aromatic PKS/T3PKS-like chemistry, and a low-confidence brefeldin-like candidate intersection (**Table 1**). These correspondences do not constitute definitive structure-BGC assignments; rather, they identify high-priority candidates where genomic potential and metabolomic evidence converge.

3.8 Medium-dependent metabolite responses of *H. finlandica*

Medium composition strongly shaped the extracellular chemical footprint of *H. finlandica*, with distinct metabolite classes preferentially enriched in GMM versus PDB cultures. In the aqueous extract, GMM cultures showed higher relative abundance of several redox-active and stress-associated putative annotations, including anthracene-like features (e.g., 3,8-dihydroxy-1-methylanthraquinone-2-carboxylic acid), glycerophospholipids (e.g., glycerophosphocholine), nucleosides (e.g., adenosine), and isocoumarin-related compounds such as desmethyldiaportinol (**Fig. 5A**). In contrast, PDB cultures showed higher abundances of alkaloids (e.g., tetrahydroharman-3-carboxylic acid), prenol lipids (e.g., caryophyllene oxide), and phenylpropanoid/polyketide-related features. Similar trends were observed in the organic extract (**Fig. 5B**). GMM cultures were enriched in redox-active naphthalenes (e.g., 2,7-dihydroxynaphthalene) and phenylpropanoid/polyketide-related features, whereas PDB cultures showed elevated nitrogen-containing alkaloid-like features such as 1,1-dimethyl-

tetrahydrocarboline-3-carboxylic acid and steroid-related annotations such as allotetrahydrocortisol. Because residual nutrients were not measured at the end of cultivation these differences are interpreted as medium-associated metabolite responses rather than evidence of nutrient depletion or physiological nutrient limitation.

3.9 Contextualizing *H. finlandica* metabolites using community repositories

Public metabolomics repositories enable placement of detected metabolites within broader taxonomic and ecological contexts. Using microbeMASST (68), we queried representative *H. finlandica* metabolites against publicly available datasets to assess their distribution across microbial lineages. Although limited deposition of fungal endophyte metabolomes constrains phylogenetic resolution, several informative patterns emerged. A diterpene, 7-oxoabieta-8,11,13-trien-18-oic acid feature, supported by authentic-standard MS/MS comparison, matched spectra from *Streptomyces* spp. and fungi including *Tolypocladium* sp. (**Supplemental Fig. 1A**). The putative benzopyran polyketide desmethyldiaportinol showed spectral similarity primarily to *Alternaria* datasets (**Supplemental Fig. 1B**). In contrast, the siderophore desferricrocin exhibited closely matching signatures across diverse fungi, including *Chalciporus piperatus* in Basidiomycota and multiple genera (e.g., *Botrytis*, *Cladosporium, Fusarium*) in Ascomycota, consistent with broad fungal siderophore biology (**Supplemental Fig. 1C**).

These examples illustrate how community metabolomics resources can contextualize *H. finlandica* metabolites within the broader fungal chemical landscape. They also reinforce the need for conservative interpretation: database co-occurrence and spectral similarity can support prioritization and ecological context, but require authentic standards, NMR, targeted genetics, isotope labeling, or heterologous expression for definitive structural or biosynthetic assignments.

4 Discussion

Dark septate endophytes are common in forest soils and influence plant growth, stress tolerance, and nutrient allocation under experimental conditions (10, 62, 69-74). Because curated fungal ecology resources are incomplete for many non-model taxa, including *Hyaloscypha*, we treated ecological assignments as working hypotheses derived from source literature, genome metadata, and nomenclature updates. Within this curated 41-genome panel, three complementary BGC prediction pipelines converged on enriched PKS-, NRPS-, and terpene-associated biosynthetic capacity in DSEs, although the strength of enrichment varied by tool and BGC class. Phylogenetically informed models were broadly consistent across taxonomy-based and genome-derived trees, with instability largely confined to sparsely represented classes.

The predictors used here captured overlapping but non-identical views of fungal biosynthetic potential. antiSMASH is conservative and anchored to MIBiG reference comparisons; FunBGCeX expands fungal detection, including domainless biosynthetic enzymes; and BGC-Prophet applies protein language modeling and yielded higher cluster counts while preserving rank concordance across genomes. Product-label agreement across tools was limited, but genome-level rank agreement was strong. This supports convergence on relative biosynthetic capacity, not on exact chemical products. Accordingly named antiSMASH or FunBGCeX labels are best interpreted as reference similarities unless supported by deeper domain analysis, synteny, and metabolite evidence.

The GCF analysis further supports the view that DSE genomes contain substantial, underexplored biosynthetic diversity. Most BiG-SCAPE families were restricted to a single genome, and only one GCF was shared across all seven primary ecological categories represented in the comparison. DSE genomes carried more NRPS GCFs and more total GCFs in rank-based

tests, consistent with the BGC count enrichment, but the weaker phylogenetic support indicates that ecological guild, lineage history, and uneven sampling are difficult to separate in the current dataset. Thus, the GCF results should not be interpreted as evidence for DSE-specific products; rather, they reinforce that DSE-associated biosynthetic space remains sparsely characterized and warrants broader, taxonomically balanced genome-mining paired with metabolomics.

For *H. finlandica* specifically, genome mining supported substantial PKS, NRPS, and terpene capacity. Several candidate intersections were consistent with metabolomics results. PKS/T1PKS reference similarities to DHN-melanin-related intermediates align with the biology of melanized DSEs and the detection of dihydroxynaphthalene-like features (75), but LC-MS/MS could not resolve positional isomers. The siderophore-associated NRPS candidate aligns with the detection of desferricrocin/ferricrocin-like features and with broad fungal siderophore biology (76-78). A brefeldin-like candidate remains low-confidence as BGC reference similarity score was low and the metabolite annotation remains to be structurally validated. The authentic-standard comparison for 7-oxodehydroabietic acid provides the strongest metabolite-level evidence. The standard-supported MS/MS match strengthens the biological relevance of terpene-associated chemistry in *H. finlandica*, particularly because comparative genome mining suggested enriched terpene capacity among DSE genomes.

Across extracts, enriched metabolite classes peptide-like compounds, aromatic polyketides, fatty-acid-derived/lactone features, siderophore-like features, and sesquiterpenoid-like annotations. Interpreted cautiously, these patterns are consistent with (i) NRPS/hybrid activity yielding peptide-like and siderophore-type chemistries (77, 79), (ii) NR-PKS activity producing aromatic scaffolds such as benzopyrans and naphthopyrones (80-82), (iii) HR-PKS contributions

to lipid-like and oxylipin-related features (83), and (iv) terpene synthases generating sesquiterpenoids, often partnered with oxidative tailoring enzymes (84).

These results align with prior Helotiales observations. *Sclerotinia sclerotiorum* encodes ~80 BGCs with infection-linked expression (85), Helotiales sp. BL73 carries extensive predicted terpene capacity (86), and *Hymenoscyphus fraxineus*/*H. albidus* show expanded repetitive content and BGC repertoires (87). Metabolomics studies likewise report peptide-like compounds, cholines, nucleobases/nucleotides, sugars, and siderophores in *Pinus sylvestris*-associated DSEs (88) and polyketide-rich chemistry in halophyte DSEs such as *Gaeumannomyces* sp. (89).

A persistent interpretive limitation in untargeted metabolomics is separating de novo fungal metabolites from medium-derived compounds and transformation products, enzymatic transformation products, and intracellular metabolites release by leakage or partial lysis during growth and processing. Both PDB and GMM contain substrates that can be assimilated or enzymatically remodeled. The exometabolome measured here is therefore best interpreted as an integrated extracellular footprint of fungal growth under defined media contexts, not as a catalog of terminal biosynthetic products (28, 90). Nonetheless, consistent enrichment relative to matched uninoculated controls and strong separation in ordination space indicate that *H. finlandica* reproducibly reshapes its extracellular chemical environment.

In summary, this analysis establishes *H. finlandica* PMI 746 as a paired genome-mining and exometabolomics reference for DSE metabologenomics. The dataset supports candidate links among encoded PKS, NRPS, terpene, and siderophore capacity and observed extracellular chemical features, while explicitly distinguishing prediction, putative annotation, standard-supported metabolite evidence, and unresolved BGC-product assignment. Definitive genome-metabolite links will require targeted genetics, heterologous expression, isotope labeling, purified

standards, and/or NMR. The public datasets and code are intended to support community-driven exploration of DSE specialized metabolism without over-assigning function in a context-dependent fungal guild.

Acknowledgements

This research was funded by the Genomic System Sciences Program, U.S. Department of Energy, Office of Science, Biological and Environmental Research, as part of the Plant-Microbe Interfaces Scientific Focus Area (ORNL; https://pmiweb.ornl.gov/). Oak Ridge National Laboratory is managed by UT-Battelle, LLC., for the US DOE under contract No. DE-AC05-00OR22725.

Competing interests

None declared.

Author contributions

T.A.R. and P.E.A. created the experimental design. T.A.R and J.B.C. conducted the experiments to induce and extract metabolites from *Hyaloscypha finlandica* and nutrient media. S.M. and P.E.A. prepared samples for liquid chromatography-mass spectrometry and performed UPLC-ESI-MS/MS analysis. J.B.C. and J.A.O. performed the genome-mining procedure and statistical analyses. J.B.C., S.M., P.E.A., and T.A.R. wrote the manuscript with contributions from all co-authors.

Data availability

Raw LC-MS/MS data are available through the MassIVE repository under accession MSV000099765 and the Paired Omics Data Platform (PODP) data are recorded at https://pairedomicsdata.bioinformatics.nl/projects/deffa5a6-9d05-4b8f-b12a-fc8fa7a57e76.2. Processed feature tables, annotation tables with confidence levels, genome-mining summaries, GCF outputs, and analysis scripts are available at https://doi.org/10.25983/PMI/3366812.

Declaration of generative AI and AI-assisted technologies in the writing process

During the preparation of this work, the authors used ChatGPT to improve readability and editing of the manuscript, but not for data generation, analysis, references or substantive writing. After using this tool/service, the authors reviewed and edited the content as needed and take full responsibility for the content of the publication.

Table 1. Representative similarities between predicted biosynthetic gene clusters (BGCs) and LC-MS/MS compound annotations in *Hyaloscypha finlandica* PMI 746. Shown are putative compound-level intersections identified by reference-based genome mining tools and matched to spectral libraries.

Tool	BGC Class	Genomic Prediction Top Candidate	Tool Confidence Score	Metabolomic Prediction Top Candidate	Spectral match Confidence Score[1]
antiSMASH	PKS/T1PKS	1,3,6,8-tetrahydroxynaphthalene	High	Dihydroxynaphthalene[3]	88
antiSMASH	PKS/T1PKS	4-epi-15-epi-brefeldin A	Low	Brefeldin C	78
antiSMASH[2]	NRPS	Triacetylfusarinine C or hydroxyferricrocin	High	Desferricrocin	79
FungBGCeX	NRPS or NRPS-like	choline	High	Glycerophosphocholine	92
FungBGCeX	PKS/T3PKS	Alkyl pyrones, resorcylic acids, resorcinols	Med	Resorcinol-O,O′-diacetic acid[4]	77

[1]Spectral match confidence using NIST 2023 HighChem HighRes score (low=0; high=100)

[2]This compound was also identified by the FungBGCeX tool.

[3]The positional isomers of the two hydroxyl groups could not be resolved using LC-MS/MS.

[4]The detected putative compound corresponds to the dehydrated protonated ion $[M + H - H_2O]$.

Figure 1. Comparative genome mining and biosynthetic potential across 41 ascomycete fungi and related taxa. (**A**) The genome dataset of 41 ascomycete and closely related taxa was visualized in the Interactive Tree of Life (iTOL) using an NCBI taxonomy-based tree (.nwk). The phylogeny was annotated with 10 canonicalized biosynthetic gene cluster (BGC) classes—NRPS, PKS, RiPP, Terpene, Indole, Alkaloid, Saccharide, Hybrid, Other, and Unknown. Ecological metadata (i.e. inner to outer ring), including fungal class (outer shaded background) and strain-specific source-published ecology (primary and secondary assignments), are displayed as the first and second concentric rings. The third ring represents genome size (Mb), scaled from light = small to dark = large. Rings 4-6 show per-genome BGC counts by class predicted by antiSMASH, FunBGCeX, and BGC-Prophet, respectively. (**B**) Mean composition (%) of BGCs per genome by prediction tool, displayed as stacked bars normalized by total predicted clusters per genome. (**C**) Mean number of predicted BGCs per genome by fungal class and tool, showing lineage-specific enrichment of polyketide (PKS) and peptide (NRPS) clusters. (**D**) Mean composition of BGCs per genome by ecological function and tool. (**E**) Genome-specific BGC-class profiles for *Hyaloscypha finlandica* PMI 746, denoted by the green box/star in (**A**).

Figure 2. Genomes of dark septate endophytes (DSE) encode for some biosynthetic gene cluster (BGC) classes more than other related ascomycetes across three mining tools. Stacked box-and-whisker plots per tool show the distribution of per-genome BGC counts for each predicted class in Other (light; $n = 22$) and DSE (dark; $n = 19$) fungi. Brackets denote DSE versus Other comparisons and significance is represented by single ($p < 0.05$) or double ($p < 0.01$) symbols: Wilcoxon (Benjamini-Hochberg correction within tool (orange asterisks)); PGLS (phylogeny-aware (blue star)); PGLS (taxonomy-aware (magenta star)). These statistics are presented as exploratory within a reference dataset; differences across tools reflect

methodological and model-awareness variation rather than guild representation or a benchmark of tool performance.

Figure 3. Comparative distribution of predicted biosynthetic products across ecological guilds reveals conserved repertoires by genome-mining tools. antiSMASH and FunBGCeX predictions of "Most similar" secondary metabolites are shown as independent bubble plots to illustrate tool-specific ecological patterns. Each panel depicts the top 30 (top to bottom) putative compounds per ecological guild following exclusion of guilds represented by fewer than three genomes per tool. Bubble diameter and color gradient correspond to the proportion of genomes within each guild that contain ≥ 1 biosynthetic gene cluster (BGC) assigned to that compound. Compound name color corresponds to predicted BGC class (NRPS, PKS, RiPP, Terpene, Hybrid, Other). Compound names were standardized using a canonicalization pipeline that removed stereochemical prefixes, punctuation inconsistencies, and applied both a manual synonym map (e.g., (R)-mellein, (-)-mellein, mellein derivative = mellein) and fuzzy string matching (Jaro-Winkler ≤ 0.10) to collapse near-identical annotations. Where multiple candidate products were listed for a single cluster (e.g., "compound A/compound B"), only the first was retained and denoted with the suffix "(s)" to indicate a putative set of analogs. To improve readability, names exceeding 30 characters were truncated with an ellipsis ("…"). Analyses were performed independently for each tool to avoid cross-platform bias in annotation vocabulary. Therefore, X-axes differ between panels and reflect the most prevalent products from reference-based matching predicted by each pipeline.

Figure 4. Chemotaxonomic profile measured from *H. finlandica* PMI 746 exudates using untargeted metabolomics. (**A**) Nonmetric multidimensional scaling (NMDS) ordinations of aqueous (left) and organic (right) LC-MS/MS feature matrices showing separation among

Hyaloscypha finlandica PMI 746 cultures grown in glucose minimal medium (GMM) or potato dextrose broth (PDB) and their corresponding uninoculated media controls. Ellipses represent 95% confidence intervals around group centroids. (**B**) Numbers of LC-MS/MS features significantly enriched in fungal exudates relative to matched uninoculated media for aqueous and organic extracts. Features were identified by one-way ANOVA followed by Tukey HSD (FDR < 0.05, Log2 fold change ≥ 2). (**C**) Chemical superclass composition of enriched features based on structural annotation. Putative annotations were assigned via spectral library matching (NIST 2023 and GNPS) and extended using SIRIUS/CANOPUS predictions, enabling classification to the ClassyFire superclass level. Stacked bars indicate the relative contribution of major chemical superclasses among annotated aqueous and organic exudate features.

Figure 5. Class level metabolite differences among nutrient medias. (**A**) Aqueous extract and (**B**) organic extract of *Hyaloscypha finlandica* PMI 746 exudates. Each point represents the estimated Log2 fold change between PDB and GMM for a class, derived from a mixed effect model (value ~ Group + (1 | Metabolite)). Points on the right of zero indicate higher abundance in PDB; points to the left indicate higher abundance in GMM. Error bars show 95% confidence intervals. Classes are grouped and ordered by superclass. The name of the most abundant metabolite within each class (highest mean Log2 intensity across sample) is shown above its point estimate. The barplot panel shows the number of metabolites contributing to each class level model. Because one class was disproportionately large, metabolite counts were truncated but a true count is displayed next to the bar. Red point: unsignificant, black point: significant. All annotations are putative.

References:

1. Jumpponen, A., and Trappe, J.M. (1998). Dark septate endophytes: A review of facultative biotrophic root-colonizing fungi. New Phytologist. 140, 295-310. https://doi.org/10.1046/j.1469-8137.1998.00265.x.
2. Rodriguez, R.J., White, J.F., Arnold, A.E., and Redman, R.S. (2009). Fungal endophytes: Diversity and functional roles. New Phytologist. 182, 314-330.
3. Knapp, D.G., Kovács, G.M., Zajta, E., Groenewald, J.Z., and Crous, P.W. (2015). Dark septate endophytic pleosporalean genera from semiarid areas. Persoonia. 35, 87-100. https://doi.org/10.3767/003158515X687669.
4. Berthelot, C., Leyval, C., Foulon, J., Chalot, M., and Blaudez, D. (2016). Plant growth promotion, metabolite production and metal tolerance of dark septate endophytes isolated from metal-polluted poplar phytomanagement sites. FEMS Microbiol. Ecol. 92, fiw144. https://doi.org/10.1093/femsec/fiw144.
5. Han, L., Zuo, Y., He, X., Hou, Y., Li, M., and Li, B. (2021). Plant identity and soil variables shift the colonisation and species composition of dark septate endophytes associated with medicinal plants in a northern farmland in China. Appl. Soil Ecol. 167, 104042. https://doi.org/10.1016/j.apsoil.2021.104042.
6. Zuo, Y., Hu, Q., Liu, J., and He, X. (2022). Relationship of root dark septate endophytes and soil factors to plant species and seasonal variation in extremely arid desert in Northwest China. Appl. Soil Ecol. 175, 104454. https://doi.org/10.1016/j.apsoil.2022.104454.

7. Malicka, M., Magurno, F., and Piotrowska-Seget, Z. (2022). Plant association with dark septate endophytes: When the going gets tough (and stressful), the tough fungi get going. Chemosphere. 302, 134830. https://doi.org/10.1016/j.chemosphere.2022.134830.
8. Cosner, J., Pandharikar, G., Tremble, K., Nash, J., Rush, T.A., Vilgalys, R., and Veneault-Fourrey, C. (2025). Fungal endophytes. Current Biology. 35, R904-R910. https://doi.org/10.1016/j.cub.2025.08.058.
9. Ahlich, K., and Sieber, T.N. (1996). The profusion of dark septate endophytic fungi in non-ectomycorrhizal fine roots of forest trees and shrubs. New Phytologist. 132, 259-270. https://doi.org/10.1111/j.1469-8137.1996.tb01845.x.
10. Alberton, O., Kuyper, T.W., and Summerbell, R.C. (2010). Dark septate root endophytic fungi increase growth of Scots pine seedlings under elevated CO2 through enhanced nitrogen use efficiency. Plant Soil, 328, 459-470. https://doi.org/10.1007/s11104-009-0125-8.
11. Chlebicki, A. (2023). Fungal endophytes of stems and roots of *Juncus trifidus* subsp. *trifidus* from Poland. Ann. Univ. Paedagogicae Cracoviensis Studia Naturae. https://doi.org/10.24917/25438832.8.1.
12. Queloz, V., Sieber, T.N., Holdenrieder, O., McDonald, B.A., and Grünig, C.R. (2011). No biogeographical pattern for a root-associated fungal species complex. Global Ecol. Biogeography. 20, 160-169. https://doi.org/10.1111/j.1466-8238.2010.00589.x.
13. Netherway, T., Bengtsson, J., Buegger, F., Fritscher, J., Oja, J., Pritsch, K., Hildebrand, F., Krab, E.J., and Bahram, M. (2024). Pervasive associations between dark septate endophytic fungi with tree root and soil microbiomes across Europe. Nat. Commun. 15, 159. https://doi.org/10.1038/s41467-023-44172-4.

14. Nash, J., Tremble, K., Schadt, C., Cregger, M.A., Bryan, C., and Vilgalys, R. (2025). Time-series RNA metabarcoding of the active *Populus tremuloides* root microbiome reveals hidden temporal dynamics and dormant core members. mSystems. 10, e00285-25. https://doi.org/10.1128/msystems.00285-25.

15. Wang, C.J.K., and Wilcox, H.E. (1985). New species of ectendomycorrhizal and pseudomycorrhizal fungi: *Phialophora finlandia*, *Chloridium paucisporum*, and *Phialocephala fortinii*. Mycologia. 77, 951-958. https://doi.org/10.2307/3793308.

16. Harrington, T.C., and McNew, D.L. (2003). Phylogenetic analysis places the *Phialophora*-like anamorph genus *Cadophora* in the Helotiales. Mycotaxon. 87, 141-151. https://doi.org/10.5962/p.418402.

17. Fehrer, J., Réblová, M., Bambasová, V., and Vohník, M. (2019). The root-symbiotic *Rhizoscyphus ericae* aggregate and *Hyaloscypha* (Leotiomycetes) are congeneric: Phylogenetic and experimental evidence. Stud. Mycol. 92, 195-225. https://doi.org/10.1016/j.simyco.2018.10.004.

18. Ursic, M., and Peterson, R.L. (1997). Morphological and anatomical characterization of ectomycorrhizas and ectendomycorrhizas on *Pinus strobus* seedlings in a southern Ontario nursery. Can. J. Bot. 75, 2057-2072. https://doi.org/10.1139/b97-917.

19. Vrålstad, T., Schumacher, T., and Taylor, A.F.S. (2002). Mycorrhizal synthesis between fungal strains of the *Hymenoscyphus ericae* aggregate and potential ectomycorrhizal and ericoid hosts. New Phytologist. 153, 143-152. https://doi.org/10.1046/j.0028-646X.2001.00290.x.

20. Villarreal-Ruiz, L., Anderson, I.C., and Alexander, I.J. (2004). Interaction between an isolate from the *Hymenoscyphus ericae* aggregate and roots of *Pinus* and *Vaccinium*. New Phytologist. 164, 183-192. https://doi.org/10.1111/j.1469-8137.2004.01167.x.

21. Hambleton, S., and Sigler, L. (2005). *Meliniomyces*, a new anamorph genus for root-associated fungi with phylogenetic affinities to *Rhizoscyphus ericae* (*Hymenoscyphus ericae*), Leotiomycetes. Stud. Mycology. 53, 1-27. https://doi.org/10.3114/sim.53.1.1.

22. Gorfer, M., Klaubauf, S., Bandian, D., and Strauss, J. (2007). *Cadophora finlandia* and *Phialocephala fortinii*: *Agrobacterium*-mediated transformation and functional GFP expression. Mycol. Res. 111, 850-855. https://doi.org/10.1016/j.mycres.2007.05.002.

23. Mrnka, L., Tokárová, H., Vosátka, M., and Matějka, P. (2009). Interaction of soil filamentous fungi affects needle composition and nutrition of Norway spruce seedlings. Trees. 23, 887-897. https://doi.org/10.1007/s00468-009-0330-3.

24. Day, M.J., Hall, J.C., and Currah, R.S. (2012). Phialide arrangement and character evolution in the helotialean anamorph genera *Cadophora* and *Phialocephala*. Mycologia. 104, 371-381. https://doi.org/10.3852/11-059.

25. Wilcox, H.E., and Wang, C.J.K. (1987). Ectomycorrhizal and ectendomycorrhizal associations of *Phialophora finlandia* with *Pinus resinosa*, *Picea rubens*, and *Betula alleghaniensis*. Can. J. For. Res. 17, 976-990. https://doi.org/10.1139/x87-152.

26. Kernaghan, G., Sigler, L., and Khasa, D. (2003). Mycorrhizal and root endophytic fungi of containerized *Picea glauca* seedlings assessed by rDNA sequence analysis. Microb. Ecol. 45, 128-136. https://doi.org/10.1007/s00248-002-1024-1.

27. Dos Santos Utmazian, M.N., Schweiger, P., Sommer, P., Gorfer, M., Strauss, J., and Wenzel, W.W. (2007). Influence of *Cadophora finlandica* and other microbial treatments

on cadmium and zinc uptake in willows grown on polluted soil. Plant, Soil and Env. 53, 158-166. https://doi.org/10.17221/2310-PSE.

28. Brakhage, A.A. (2013). Regulation of fungal secondary metabolism. Nat. Rev. Microbiol. 11, 21-32. https://doi.org/10.1038/nrmicro2916.

29. Macheleidt, J., Mattern, D.J., Fischer, J., Netzker, T., Weber, J., Schroeckh, V., Valiante, V., and Brakhage, A.A. (2016). Regulation and role of fungal secondary metabolites. Ann. Rev. Genetics. 50, 371-392. https://doi.org/10.1146/annurev-genet-120215-035203.

30. Keller, N.P. (2019). Fungal secondary metabolism: regulation, function and drug discovery. Nat. Rev. Microbiol. 17, 167-180. https://doi.org/10.1038/s41579-018-0121-1.

31. Elhamouly, N.A., Hewedy, O.A., Zaitoon, A., Miraples, A., Elshorbagy, O.T., Hussien, S., El-Tahan, A., and Peng, D. (2022). The hidden power of secondary metabolites in plant-fungi interactions and sustainable phytoremediation. Front. Plant Sci. 13. https://doi.org/10.3389/fpls.2022.1044896.

32. Medema, M.H., and Fischbach, M.A. (2015). Computational approaches to natural product discovery. Nat. Chem. Biol. 11, 639-648. https://doi.org/10.1038/nchembio.1884.

33. Robey, M.T., Caesar, L.K., Drott, M.T., Keller, N.P., and Kelleher, N.L. (2021). An interpreted atlas of biosynthetic gene clusters from 1,000 fungal genomes. Proc. Nat. Acad. Sci. 118, e2020230118. https://doi.org/10.1073/pnas.2020230118.

34. Riedling, O., Walker, A.S., and Rokas, A. (2024). Predicting fungal secondary metabolite activity from biosynthetic gene cluster data using machine learning. Microbiol. Spectrum. 12, e03400-23. https://doi.org/10.1128/spectrum.03400-23.

35. Riedling, O.L., and Rokas, A. (2025). mGem: How many fungal secondary metabolites are produced by filamentous fungi? Conservatively, at least 1.4 million. mBio. 16, e01381-25. https://doi.org/10.1128/mbio.01381-25.
36. Knapp, D.G., and Kovács, G.M. (2016). Interspecific metabolic diversity of root-colonizing endophytic fungi revealed by enzyme activity tests. FEMS Microbiol. Ecol. 92, fiw190. https://doi.org/10.1093/femsec/fiw190.
37. Ibrahim, A., Tanney, J.B., Fei, F., Seifert, K.A., Cutler, G.C., Capretta, A., Miller, J.D., and Sumarah, M.W. (2020). Metabolomic-guided discovery of cyclic nonribosomal peptides from *Xylaria ellisii* sp. nov., a leaf and stem endophyte of *Vaccinium angustifolium*. Sci. Rep. 10, 4599. https://doi.org/10.1038/s41598-020-61088-x.
38. DesRochers, N., Renaud, J.B., Tanney, J.B., Ibrahim, A., Yeung, K.K.-C., and Sumarah, M.W. (2023). Non-targeted screening of natural products from 288 fungal endophytes from Canadian fruit crops. ACS Omega. 8, 24561-24572. https://doi.org/10.1021/acsomega.3c02786.
39. Mudbhari, S., Lofgren, L., Appidi, M.R., Vilgalys, R., Hettich, R.L., and Abraham, P.E. (2024). Decoding the chemical language of *Suillus* fungi: Genome mining and untargeted metabolomics uncover terpene chemical diversity. mSystems. 9, e01225-23. https://doi.org/10.1128/msystems.01225-23.
40. Dührkop, K., Fleischauer, M., Ludwig, M., Aksenov, A.A., Melnik, A.V., Meusel, M., Dorrestein, P.C., Rousu, J., and Böcker, S. (2019). SIRIUS 4: a rapid tool for turning tandem mass spectra into metabolite structure information. Nat. Methods. 16, 299-302. https://doi.org/10.1038/s41592-019-0344-8.

41. Dührkop, K., Nothias, L.-F., Fleischauer, M., Reher, R., Ludwig, M., Hoffmann, M.A., Petras, D., Gerwick, W.H., Rousu, J., Dorrestein, P.C., et al. (2021). Systematic classification of unknown metabolites using high-resolution fragmentation mass spectra. Nat. Biotechnol. 39, 462-471. https://doi.org/10.1038/s41587-020-0740-8.

42. Aron, A.T., Gentry, E.C., McPhail, K.L., Nothias, L.-F., Nothias-Esposito, M., Bouslimani, A., Petras, D., Gauglitz, J.M., Sikora, N., Vargas, F., et al. (2020). Reproducible molecular networking of untargeted mass spectrometry data using GNPS. Nat. Protoc. 15, 1954-1991. https://doi.org/10.1038/s41596-020-0317-5.

43. Schmid, R., Petras, D., Nothias, L.-F., Wang, M., Aron, A.T., Jagels, A., Tsugawa, H., Rainer, J., Garcia-Aloy, M., Dührkop, K., et al. (2021). Ion identity molecular networking for mass spectrometry-based metabolomics in the GNPS environment. Nat. Commun. 12, 3832. https://doi.org/10.1038/s41467-021-23953-9.

44. Eldjárn, G.H., Ramsay, A., Hooft, J.J.J. van der, Duncan, K.R., Soldatou, S., Rousu, J., Daly, R., Wandy, J., and Rogers, S. (2021). Ranking microbial metabolomic and genomic links in the NPLinker framework using complementary scoring functions. PLOS Comput. Biol. 17, e1008920. https://doi.org/10.1371/journal.pcbi.1008920.

45. Caesar, L.K., Butun, F.A., Robey, M.T., Ayon, N.J., Gupta, R., Dainko, D., Bok, J.W., Nickles, G., Stankey, R.J., Johnson, D., et al. (2023). Correlative metabologenomics of 110 fungi reveals metabolite-gene cluster pairs. Nat. Chem. Biol. 19, 846-854. https://doi.org/10.1038/s41589-023-01276-8.

46. Adejor, J., Tumukunde, E., Li, G., Shehu, T.A., Wu, L., Jiang, Z., and Wang, S. (2025). Stepping out of the dark: How metabolomics shed light on fungal biology. FEMS Microbiol. Rev. 49, fuaf028. https://doi.org/10.1093/femsre/fuaf028.

47. Siless, G.E., Gallardo, G.L., Rodriguez, M.A., Rincón, Y.A., Godeas, A.M., and Cabrera, G.M. (2018). Metabolites from the dark septate endophyte *Drechslera* sp. evaluation by LC/MS and principal component analysis of culture extracts with histone deacetylase inhibitors. Chemistry & Biodiversity. 15, e1800133. https://doi.org/10.1002/cbdv.201800133.

48. Xiaoshuang, S., Wenjing, Y., Kai, M., Qi, Z., Xun, D., Yanhong, L., and Ruifeng, J. (2021). Metabonomics analysis of dark septate endophytes *Phialocephala bamuru* A024 under the induction of *Rhizoctonia solani* Kühn. Chin. Agric. Sci. Bull. 37, 25-32. https://doi.org/10.11924/j.issn.1000-6850.casb2020-0190.

49. Wang, S., Bi, Y., Quan, W., and Christie, P. (2022). Growth and metabolism of dark septate endophytes and their stimulatory effects on plant growth. Fungal Biology. 126, 674-686. https://doi.org/10.1016/j.funbio.2022.08.006.

50. Bi, Y., Wang, S., Song, Y., Tan, H., and Christie, P. (2025). Contributions of the dark septate endophyte extracellular metabolites to plant growth, metabolism and root-associated microbial community structure. Plant Soil. 511, 189-208. https://doi.org/10.1007/s11104-024-06980-5.

51. Knapp, D.G., Németh, J.B., Barry, K., Hainaut, M., Henrissat, B., Johnson, J., Kuo, A., Lim, J.H.P., Lipzen, A., Nolan, M., et al. (2018). Comparative genomics provides insights into the lifestyle and reveals functional heterogeneity of dark septate endophytic fungi. Sci. Rep. 8, 6321. https://doi.org/10.1038/s41598-018-24686-4.

52. Bovio, E., Rancurel, C., Seassau, A., Magliano, M., Gislard, M., Loisier, A., Kuchly, C., Ponchet, M., Danchin, E.G.J., and Van Ghelder, C. (2023). Genome sequence and

annotation of *Periconia digitata* a hopeful biocontrol agent of phytopathogenic oomycetes. Sci. Data. 10, 583. https://doi.org/10.1038/s41597-023-02440-4.

53. Bonito, G., Hameed, K., Ventura, R., Krishnan, J., Schadt, C.W., and Vilgalys, R. (2016). Isolating a functionally relevant guild of fungi from the root microbiome of *Populus*. Fungal Ecol. 22, 35-42. https://doi.org/10.1016/j.funeco.2016.04.007.

54. Kaminskyj, S. (2001). Fundamentals of growth, storage, genetics and microscopy of *Aspergillus nidulans*. Fungal Genetics Rep. 48, 25-31. https://doi.org/10.4148/1941-4765.1175.

55. Rush, T.A., Wymore, A.M., Rodríguez, Jr, M., Jawdy, S., Vilgalys, R.J., Martin, M.Z., and Andrews, H.B. (2024). Fungal elemental profiling unleashed through rapid laser-induced breakdown spectroscopy (LIBS). mSystems. 9, e00919-24. https://doi.org/10.1128/msystems.00919-24.

56. Wang, M., Carver, J.J., Phelan, V.V., Sanchez, L.M., Garg, N., Peng, Y., Nguyen, D.D., Watrous, J., Kapono, C.A., Luzzatto-Knaan, T., et al. (2016). Sharing and community curation of mass spectrometry data with Global Natural Products Social Molecular Networking. Nat. Biotechnol. 34, 828-837. https://doi.org/10.1038/nbt.3597.

57. Böcker, S., Letzel, M.C., Lipták, Z., and Pervukhin, A. (2009). SIRIUS: decomposing isotope patterns for metabolite identification. Bioinformatics. 25, 218-224. https://doi.org/10.1093/bioinformatics/btn603.

58. Feunang, Y.D., Eisner, R., Knox, C., Chepelev, L., Hastings, J., Owen, G., Fahy, E., Steinbeck, C., Subramanian, S., Bolton, E., et al. (2016). ClassyFire: automated chemical classification with a comprehensive, computable taxonomy. J. Cheminform. 8, 61. https://doi.org/10.1186/s13321-016-0174-y.

59. Sumner, L.W., Amberg, A., Barrett, D., Beale, M.H., Beger, R., Daykin, C.A., Fan, T.W.-M., Fiehn, O., Goodacre, R., Griffin, J.L., et al. (2007). Proposed minimum reporting standards for chemical analysis Chemical Analysis Working Group (CAWG) Metabolomics Standards Initiative (MSI). Metabolomics 3, 211-221. https://doi.org/10.1007/s11306-007-0082-2.

60. Schymanski, E.L., Jeon, J., Gulde, R., Fenner, K., Ruff, M., Singer, H.P., and Hollender, J. (2014). Identifying small molecules via high resolution mass spectrometry: Communicating confidence. Environ. Sci. Technol. 48, 2097-2098. https://doi.org/10.1021/es5002105.

61. Grünig, C.R., Queloz, V., and Sieber, T.N. (2011). Structure of Diversity in Dark Septate Endophytes: From Species to Genes. In Endophytes of Forest Trees: Biology and Applications, A. M. Pirttilä and A. C. Frank, eds. Springer Netherlands. pp. 3-30. https://doi.org/10.1007/978-94-007-1599-8_1.

62. Newsham, K.K. (2011). A meta-analysis of plant responses to dark septate root endophytes. New Phytologist. 190, 783-793. https://doi.org/10.1111/j.1469-8137.2010.03611.x.

63. Santos, M., Cesanelli, I., Diánez, F., Sánchez-Montesinos, B., and Moreno-Gavíra, A. (2021). Advances in the role of dark septate endophytes in the plant resistance to abiotic and biotic stresses. J. Fungi. 7, 939. https://doi.org/10.3390/jof7110939.

64. Blin, K., Shaw, S., Vader, L., Szenei, J., Reitz, Z.L., Augustijn, H.E., Cediel-Becerra, J.D.D., de Crécy-Lagard, V., Koetsier, R.A., Williams, S.E., et al. (2025). antiSMASH 8.0: extended gene cluster detection capabilities and analyses of chemistry, enzymology, and regulation. Nucleic Acids Res. 53, W32-W38. https://doi.org/10.1093/nar/gkaf334.

65. Tang, J., and Matsuda, Y. (2024). Discovery of fungal onoceroid triterpenoids through domainless enzyme-targeted global genome mining. Nat. Commun. 15, 4312. https://doi.org/10.1038/s41467-024-48771-7.

66. Lai, Q., Yao, S., Zha, Y., Zhang, H., Zhang, H., Ye, Y., Zhang, Y., Bai, H., and Ning, K. (2025). Deciphering the biosynthetic potential of microbial genomes using a BGC language processing neural network model. Nuc. Acids Res. 53, gkaf305. https://doi.org/10.1093/nar/gkaf305.

67. Katz, L.S., Griswold, T., Morrison, S.S., Caravas, J.A., Zhang, S., Bakker, H.C. den, Deng, X., and Carleton, H.A. (2019). Mashtree: A rapid comparison of whole genome sequence files. J. Open Source Soft. 4, 1762. https://doi.org/10.21105/joss.01762.

68. Zuffa, S., Schmid, R., Bauermeister, A., P. Gomes, P.W., Caraballo-Rodriguez, A.M., El Abiead, Y., Aron, A.T., Gentry, E.C., Zemlin, J., Meehan, M.J., et al. (2024). microbeMASST: a taxonomically informed mass spectrometry search tool for microbial metabolomics data. Nat. Microbiol. 9, 336-345. https://doi.org/10.1038/s41564-023-01575-9.

69. Tejesvi, M.V., Ruotsalainen, A.L., Markkola, A.M., and Pirttilä, A.M. (2010). Root endophytes along a primary succession gradient in northern Finland. Fungal Diversity. 41, 125-134. https://doi.org/10.1007/s13225-009-0016-6.

70. Hazard, C., Gosling, P., Mitchell, D.T., Doohan, F.M., and Bending, G.D. (2014). Diversity of fungi associated with hair roots of ericaceous plants is affected by land use. FEMS Microb. Ecol. 87, 586-600. https://doi.org/10.1111/1574-6941.12247.

71. An, H., Liu, Y., Zhao, X., Huang, Q., Yuan, S., Yang, X., and Dong, J. (2015). Characterization of cadmium-resistant endophytic fungi from *Salix variegata* Franch in

Three Gorges Reservoir Region, China. Microbiol. Res. 176, 29-37. https://doi.org/10.1016/j.micres.2015.03.013.

72. Hamim, A., Miché, L., Douaik, A., Mrabet, R., Ouhammou, A., Duponnois, R., and Hafidi, M. (2017). Diversity of fungal assemblages in roots of Ericaceae in two Mediterranean contrasting ecosystems. Comptes Rendus Biologies. 340, 226-237. https://doi.org/10.1016/j.crvi.2017.02.003.

73. Koizumi, T., and Nara, K. (2017). Communities of putative ericoid mycorrhizal fungi isolated from alpine dwarf shrubs in Japan: Effects of host identity and microhabitat. Microbes Env. 32, 147-153. https://doi.org/10.1264/jsme2.ME16180.

74. Daghino, S., Martino, E., Voyron, S., and Perotto, S. (2022). Metabarcoding of fungal assemblages in *Vaccinium myrtillus* endosphere suggests colonization of above-ground organs by some ericoid mycorrhizal and DSE fungi. Sci. Rep. 12, 11013. https://doi.org/10.1038/s41598-022-15154-1.

75. Eisenman, H.C., and Casadevall, A. (2012). Synthesis and assembly of fungal melanin. Appl. Microbiol. Biotechnol. 93, 931-940. https://doi.org/10.1007/s00253-011-3777-2.

76. Eisendle, M., Schrettl, M., Kragl, C., Müller, D., Illmer, P., and Haas, H. (2006). The intracellular siderophore ferricrocin is involved in iron storage, oxidative-stress resistance, germination, and sexual development in *Aspergillus nidulans*. Eukaryotic Cell. 5, 1596-1603. https://doi.org/10.1128/ec.00057-06.

77. Haas, H. (2014). Fungal siderophore metabolism with a focus on *Aspergillus fumigatus*. Nat. Prod. Rep. 31, 1266-1276. https://doi.org/10.1039/C4NP00071D.

78. Happacher, I., Aguiar, M., Yap, A., Decristoforo, C., and Haas, H. (2023). Fungal siderophore metabolism with a focus on *Aspergillus fumigatus*: Impact on biotic

interactions and potential translational applications. Essays Biochem. 67, 829-842. https://doi.org/10.1042/EBC20220252.

79. Bloudoff, K., Fage, C.D., Marahiel, M.A., and Schmeing, T.M. (2017). Structural and mutational analysis of the nonribosomal peptide synthetase heterocyclization domain provides insight into catalysis. Proc. Nat. Acad. Sci. 114, 95-100. https://doi.org/10.1073/pnas.1614191114.

80. Watanabe, A., Fujii, I., Sankawa, U., Mayorga, M.E., Timberlake, W.E., and Ebizuka, Y. (1999). Re-identification of *Aspergillus nidulans* wA gene to code for a polyketide synthase of naphthopyrone. Tetrahedron Letters. 40, 91-94. https://doi.org/10.1016/S0040-4039(98)80027-0.

81. Xu, J., Jiang, C., Zhang, Z., Ma, W., and Guo, Y. (2014). Recent progress regarding the bioactivities, biosynthesis and synthesis of naturally occurring resorcinolic macrolides. Acta Pharmacol. Sin. 35, 316-330. https://doi.org/10.1038/aps.2013.155.

82. Löhr, N.A., Rakhmanov, M., Wurlitzer, J.M., Lackner, G., Gressler, M., and Hoffmeister, D. (2023). Basidiomycete non-reducing polyketide synthases function independently of SAT domains. Fungal Bio. Biotechnol. 10, 17. https://doi.org/10.1186/s40694-023-00164-z.

83. Brodhun, F., and Feussner, I. (2011). Oxylipins in fungi. FEBS J. 278, 1047-1063. https://doi.org/10.1111/j.1742-4658.2011.08027.x.

84. González-Hernández, R.A., Valdez-Cruz, N.A., Macías-Rubalcava, M.L., and Trujillo-Roldán, M.A. (2023). Overview of fungal terpene synthases and their regulation. World J. Microbiol. Biotechnol. 39, 194. https://doi.org/10.1007/s11274-023-03635-y.

85. Graham-Taylor, C., Kamphuis, L.G., and Derbyshire, M.C. (2020). A detailed in silico analysis of secondary metabolite biosynthesis clusters in the genome of the broad host range plant pathogenic fungus *Sclerotinia sclerotiorum*. BMC Genomics. 21, 7. https://doi.org/10.1186/s12864-019-6424-4.

86. Oberhofer, M., Malfent, F., Zehl, M., Urban, E., Wackerlig, J., Reznicek, G., Vignolle, G.A., Rückert, C., Busche, T., Wibberg, D., et al. (2022). Biosynthetic potential of the endophytic fungus Helotiales sp. BL73 revealed via compound identification and genome mining. Appl. Env. Microbiol. 88, e02510-21. https://doi.org/10.1128/aem.02510-21.

87. Elfstrand, M., Chen, J., Cleary, M., Halecker, S., Ihrmark, K., Karlsson, M., Davydenko, K., Stenlid, J., Stadler, M., and Durling, M.B. (2021). Comparative analyses of the *Hymenoscyphus fraxineus* and *Hymenoscyphus albidus* genomes reveals potentially adaptive differences in secondary metabolite and transposable element repertoires. BMC Genomics. 22, 1-15. https://doi.org/10.1186/s12864-021-07837-2.

88. Tienaho, J., Karonen, M., Muilu-Mäkelä, R., Wähälä, K., Leon Denegri, E., Franzén, R., Karp, M., Santala, V., and Sarjala, T. (2019). Metabolic profiling of water-soluble compounds from the extracts of dark septate endophytic fungi (DSE) isolated from Scots pine (*Pinus sylvestris* L.) seedlings using UPLC-Orbitrap-MS. Molecules. 24, 2330. https://doi.org/10.3390/molecules24122330.

89. Lee, C., Kim, S., Li, W., Bang, S., Lee, H., Lee, H.-J., Noh, E.-Y., Park, J.-E., Bang, W.Y., and Shim, S.H. (2017). Bioactive secondary metabolites produced by an endophytic fungus *Gaeumannomyces* sp. JS0464 from a maritime halophyte *Phragmites communis*. J. Antibiot. 70, 737-742. https://doi.org/10.1038/ja.2017.39.

90. Wisecaver, J.H., and Rokas, A. (2015). Fungal metabolic gene clusters—caravans traveling across genomes and environments. Front. Microbiol. 6. https://doi.org/10.3389/fmicb.2015.00161.

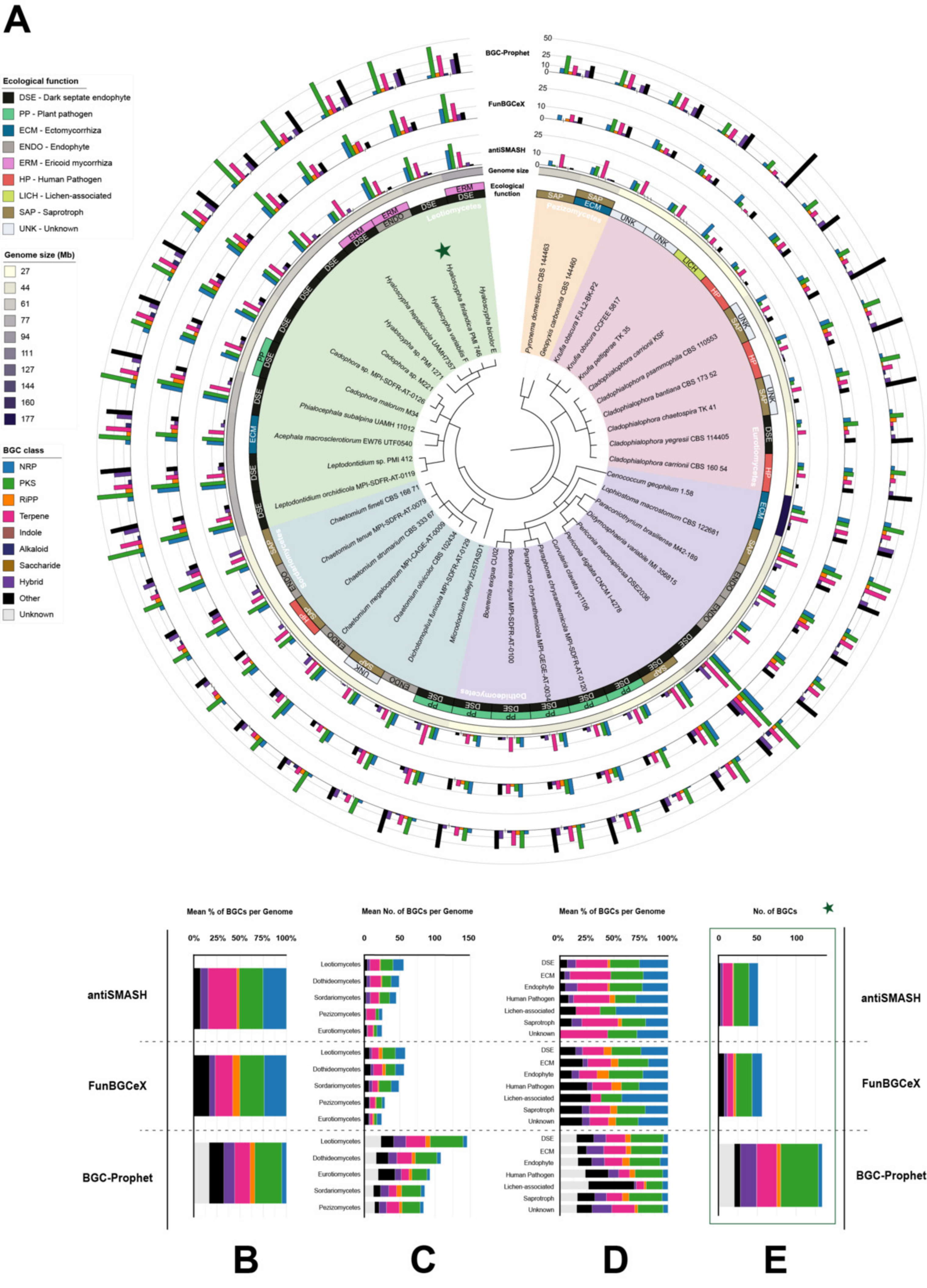
A
Ecological function
DSE - Dark septate endophyte
PP - Plant pathogen
ECM - Ectomycorrhiza
ENDO - Endophyte
ERM - Ericoid mycorrhiza
HP - Human Pathogen
LICH - Lichen-associated
SAP - Saprotroph
UNK - Unknown
Genome size (Mb)
BGC class
NRP
PKS
RiPP
Terpene
Indole
Alkaloid
Saccharide
Hybrid
Other
Unknown
BGC-Prophet
FunBGCeX
antiSMASH
Genome size
Ecological function
Leotiomycetes
Pezizomycetes
Eurotiomycetes
Dothideomycetes
Sordariomycetes
Mean % of BGCs per Genome
Mean No. of BGCs per Genome
No. of BGCs
B
C
D
E

Mean # of BGCs by Class per Genome

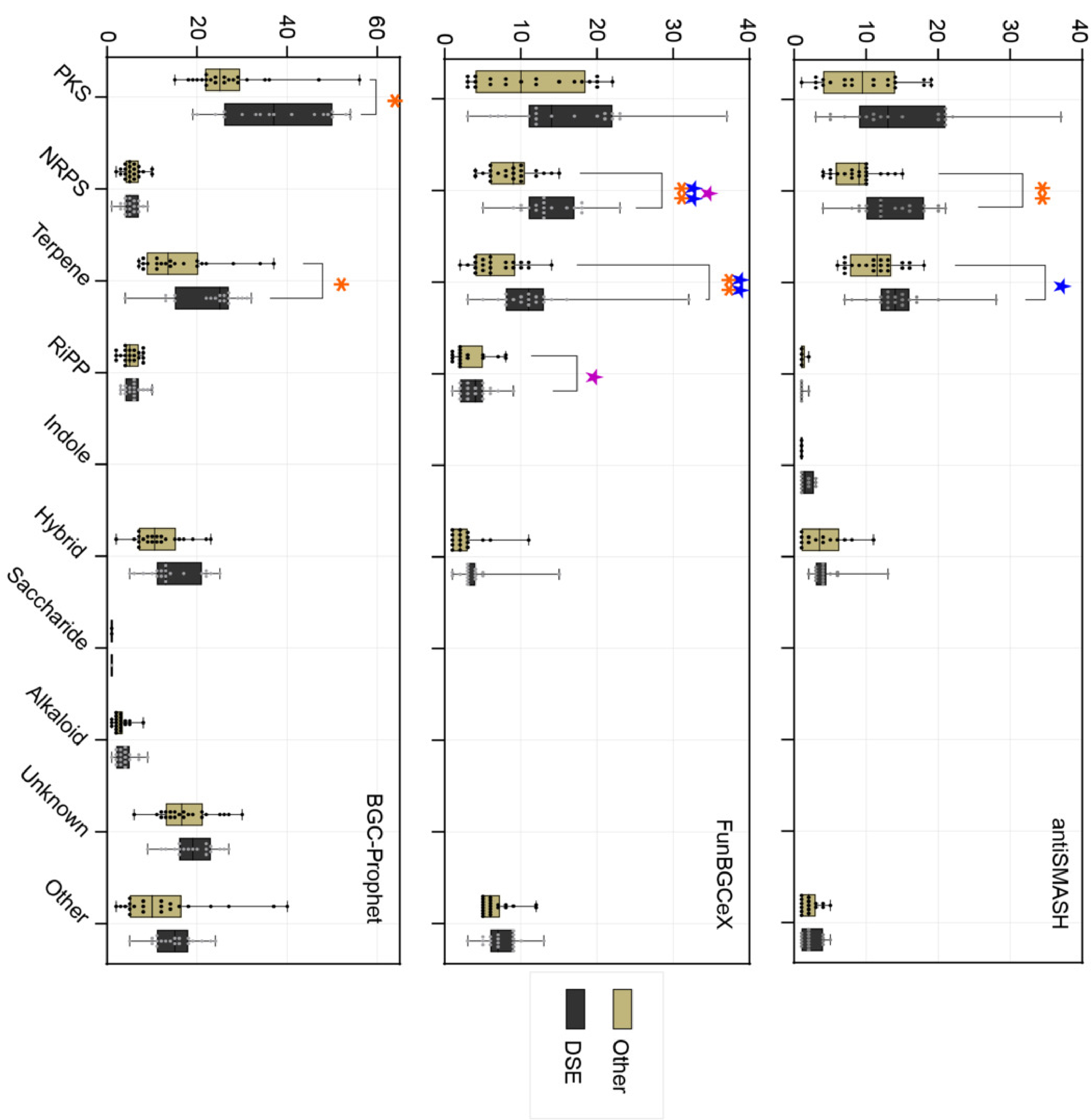

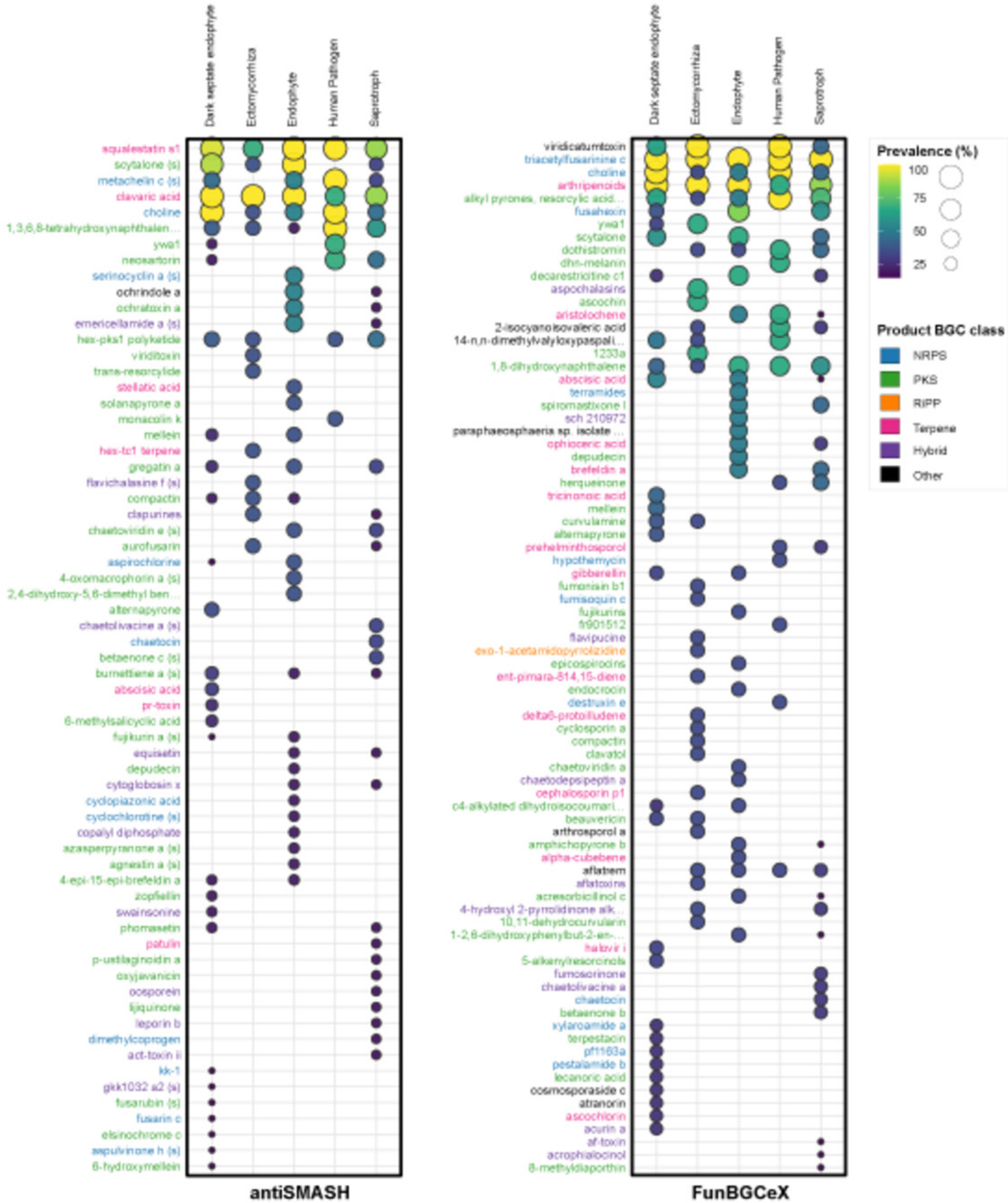

Dark septate endophyte
Ectomycorrhiza
Endophyte
Human Pathogen
Saprotroph
squalestatin s1
scytalone (s)
metachelin c (s)
clavaric acid
choline
1,3,6,8-tetrahydroxynaphthalen…
ywa1
neosartorin
serinocyclin a (s)
ochrindole a
ochratoxin a
emericellamide a (s)
hex-pks1 polyketide
viriditoxin
trans-resorcylide
stellatic acid
solanapyrone a
monacolin k
mellein
hex-tc1 terpene
gregatin a
flavichalasine f (s)
compactin
clapurines
chaetoviridin e (s)
aurofusarin
aspirochlorine
4-oxomacrophorin a (s)
2,4-dihydroxy-5,6-dimethyl ben…
alternapyrone
chaetolivacine a (s)
chaetocin
betaenone c (s)
burnettiene a (s)
abscisic acid
pr-toxin
6-methylsalicylic acid
fujikurin a (s)
equisetin
depudecin
cytoglobosin x
cyclopiazonic acid
cyclochlorotine (s)
copalyl diphosphate
azasperpyranone a (s)
agnestin a (s)
4-epi-15-epi-brefeldin a
zopfiellin
swainsonine
phomasetin
patulin
p-ustilaginoidin a
oxyjavanicin
oosporein
lijiquinone
leporin b
dimethylcoprogen
act-toxin ii
kk-1
gkk1032 a2 (s)
fusarubin (s)
fusarin c
elsinochrome c
aspulvinone h (s)
6-hydroxymellein
antiSMASH
viridicatumtoxin
triacetylfusarinine c
choline
arthripenoids
alkyl pyrones, resorcylic acid…
fusaheixin
ywa1
scytalone
dothistromin
dhn-melanin
decarestrictine c1
aspochalasins
ascochin
aristolochene
2-isocyanoisovaleric acid
14-n,n-dimethylvalyloxypaspali…
1233a
1,8-dihydroxynaphthalene
abscisic acid
terramides
spiromastixone l
sch 210972
paraphaeosphaeria sp. isolate …
ophioceric acid
depudecin
brefeldin a
herqueinone
tricinonoic acid
mellein
curvulamine
alternapyrone
prehelminthosporol
hypothemycin
gibberellin
fumonisin b1
fumisoquin c
fujikurins
fr901512
flavipucine
exo-1-acetamidopyrrolizidine
epicospirocins
ent-pimara-8(14),15-diene
endocrocin
destruxin e
delta6-protoilludene
cyclosporin a
compactin
clavatol
chaetoviridin a
chaetodepsipeptin a
cephalosporin p1
c4-alkylated dihydroisocoumari…
beauvericin
arthrosporol a
amphichopyrone b
alpha-cubebene
aflatrem
aflatoxins
acresorbicillinol c
4-hydroxyl 2-pyrrolidinone alk…
10,11-dehydrocurvularin
1-2,6-dihydroxyphenylbut-2-en-…
halovir i
5-alkenylresorcinols
fumosorinone
chaetolivacine a
chaetocin
betaenone b
xylaroamide a
terpestacin
pf1163a
pestalamide b
lecanoric acid
cosmosporaside c
atranorin
ascochlorin
acurin a
af-toxin
acrophialocinol
8-methyldiaporthin
FunBGCeX
Prevalence (%)
100
75
50
25
Product BGC class
NRPS
PKS
RiPP
Terpene
Hybrid
Other

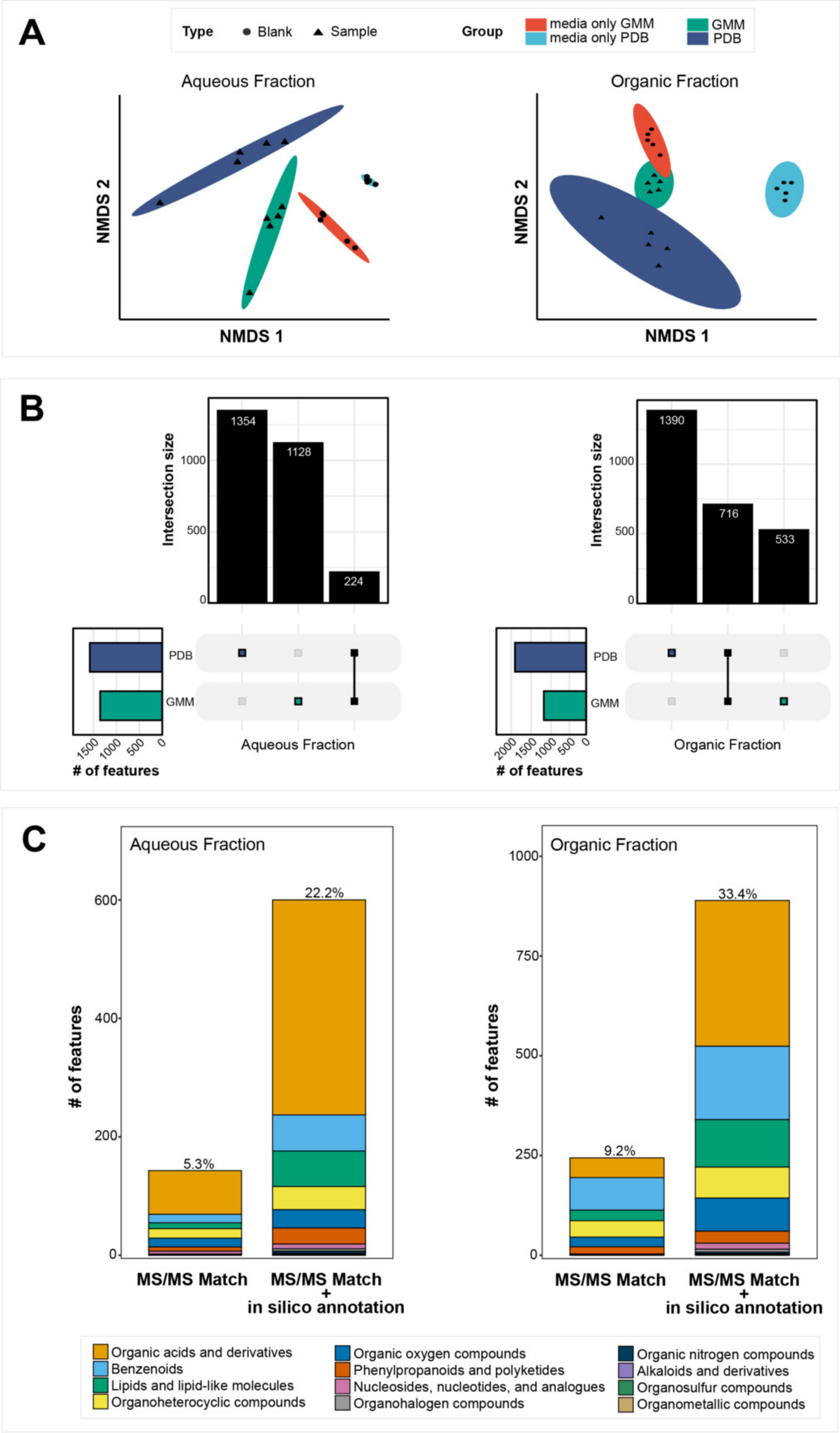
A
Type
Blank
Sample
Group
media only GMM
media only PDB
GMM
PDB
Aqueous Fraction
Organic Fraction
NMDS 2
NMDS 1
B
Intersection size
1354
1128
224
1390
716
533
PDB
GMM
Aqueous Fraction
Organic Fraction
# of features
C
Aqueous Fraction
Organic Fraction
# of features
5.3%
22.2%
9.2%
33.4%
MS/MS Match
MS/MS Match + in silico annotation
Organic acids and derivatives
Benzenoids
Lipids and lipid-like molecules
Organoheterocyclic compounds
Organic oxygen compounds
Phenylpropanoids and polyketides
Nucleosides, nucleotides, and analogues
Organohalogen compounds
Organic nitrogen compounds
Alkaloids and derivatives
Organosulfur compounds
Organometallic compounds

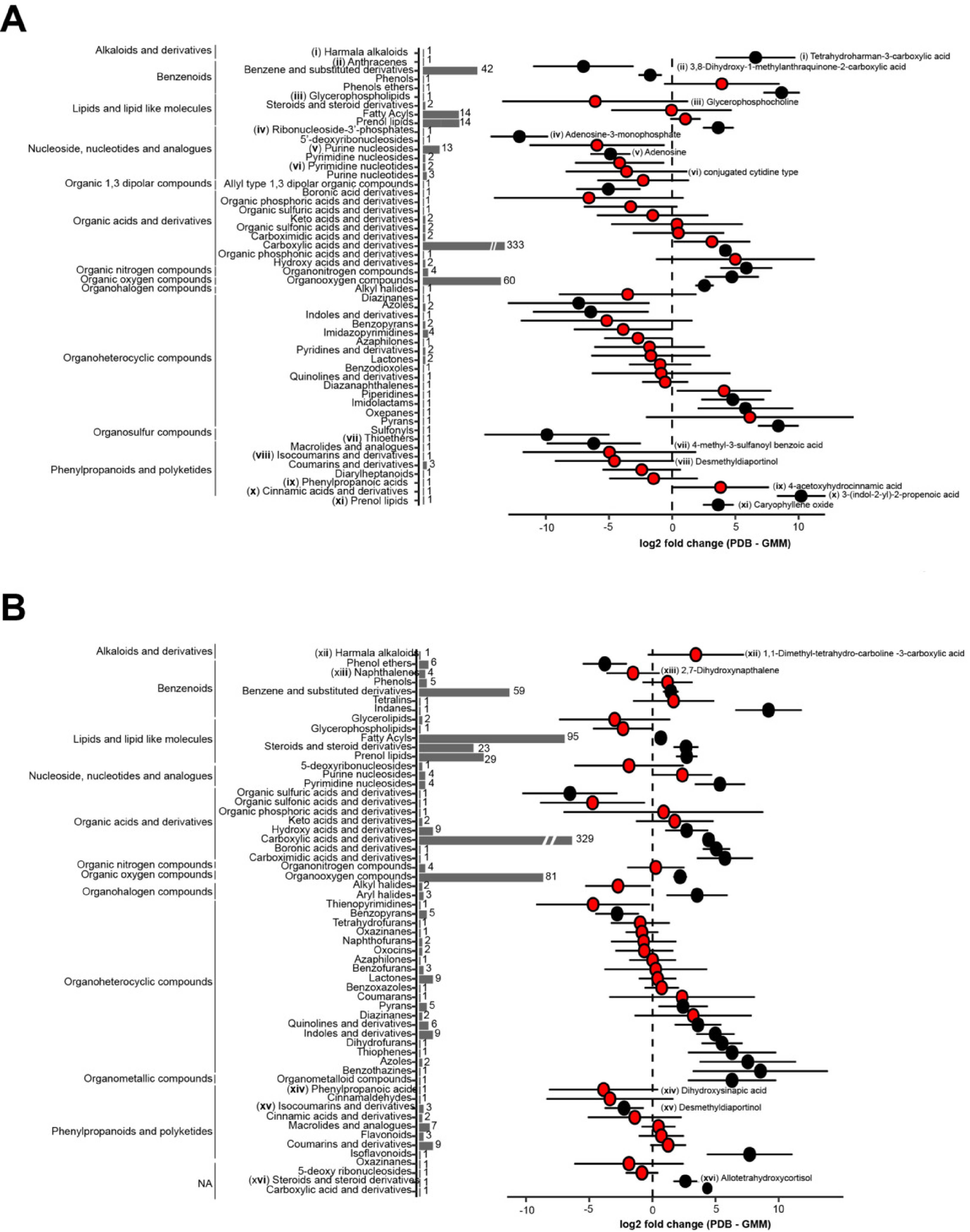
A
Alkaloids and derivatives
Benzenoids
Lipids and lipid like molecules
Nucleoside, nucleotides and analogues
Organic 1,3 dipolar compounds
Organic acids and derivatives
Organic nitrogen compounds
Organic oxygen compounds
Organohalogen compounds
Organoheterocyclic compounds
Organosulfur compounds
Phenylpropanoids and polyketides
(i) Harmala alkaloids 1
(ii) Anthracenes 1
Benzene and substituted derivatives 42
Phenols 1
Phenols ethers 1
(iii) Glycerophospholipids 1
Steroids and steroid derivatives 2
Fatty Acyls 14
Prenol lipids 14
(iv) Ribonucleoside-3'-phosphates 1
5'-deoxyribonucleosides 1
(v) Purine nucleosides 13
Pyrimidine nucleosides 2
(vi) Pyrimidine nucleotides 2
Purine nucleotides 3
Allyl type 1,3 dipolar organic compounds 1
Boronic acid derivatives 1
Organic phosphoric acids and derivatives 1
Organic sulfuric acids and derivatives 1
Keto acids and derivatives 2
Organic sulfonic acids and derivatives 2
Carboximidic acids and derivatives 2
Carboxylic acids and derivatives 333
Organic phosphonic acids and derivatives 1
Hydroxy acids and derivatives 2
Organonitrogen compounds 4
Organooxygen compounds 60
Alkyl halides 1
Diazinanes 1
Azoles 2
Indoles and derivatives 1
Benzopyrans 2
Imidazopyrimidines 4
Azaphilones 1
Pyridines and derivatives 2
Lactones 2
Benzodioxoles 1
Quinolines and derivatives 1
Diazanaphthalenes 1
Piperidines 1
Imidolactams 1
Oxepanes 1
Pyrans 1
Sulfonyls 1
(vii) Thioethers 1
Macrolides and analogues 1
(viii) Isocoumarins and derivatives 1
Coumarins and derivatives 3
Diarylheptanoids 1
(ix) Phenylpropanoic acids 1
(x) Cinnamic acids and derivatives 1
(xi) Prenol lipids 1
(i) Tetrahydroharman-3-carboxylic acid
(ii) 3,8-Dihydroxy-1-methylanthraquinone-2-carboxylic acid
(iii) Glycerophosphocholine
(iv) Adenosine-3-monophosphate
(v) Adenosine
(vi) conjugated cytidine type
(vii) 4-methyl-3-sulfanoyl benzoic acid
(viii) Desmethyldiaportinol
(ix) 4-acetoxyhydrocinnamic acid
(x) 3-(indol-2-yl)-2-propenoic acid
(xi) Caryophyllene oxide
-10
-5
0
5
10
log2 fold change (PDB - GMM)
B
Alkaloids and derivatives
Benzenoids
Lipids and lipid like molecules
Nucleoside, nucleotides and analogues
Organic acids and derivatives
Organic nitrogen compounds
Organic oxygen compounds
Organohalogen compounds
Organoheterocyclic compounds
Organometallic compounds
Phenylpropanoids and polyketides
NA
(xii) Harmala alkaloids 1
Phenol ethers 6
(xiii) Naphthalenes 4
Phenols 5
Benzene and substituted derivatives 59
Tetralins 1
Indanes 1
Glycerolipids 2
Glycerophospholipids 1
Fatty Acyls 95
Steroids and steroid derivatives 23
Prenol lipids 29
5-deoxyribonucleosides 1
Purine nucleosides 4
Pyrimidine nucleosides 4
Organic sulfuric acids and derivatives 1
Organic sulfonic acids and derivatives 1
Organic phosphoric acids and derivatives 1
Keto acids and derivatives 2
Hydroxy acids and derivatives 9
Carboxylic acids and derivatives 329
Boronic acids and derivatives 1
Carboximidic acids and derivatives 1
Organonitrogen compounds 4
Organooxygen compounds 81
Alkyl halides 2
Aryl halides 3
Thienopyrimidines 1
Benzopyrans 5
Tetrahydrofurans 1
Oxazinanes 1
Naphthofurans 2
Oxocins 2
Azaphilones 1
Benzofurans 3
Lactones 9
Benzoxazoles 1
Coumarans 1
Pyrans 5
Diazinanes 2
Quinolines and derivatives 6
Indoles and derivatives 9
Dihydrofurans 1
Thiophenes 1
Azoles 2
Benzothazines 1
Organometalloid compounds 1
(xiv) Phenylpropanoic acids 1
Cinnamaldehydes 1
(xv) Isocoumarins and derivatives 3
Cinnamic acids and derivatives 2
Macrolides and analogues 7
Flavonoids 3
Coumarins and derivatives 9
Isoflavonoids 1
Oxazinanes 1
5-deoxy ribonucleosides 1
(xvi) Steroids and steroid derivatives 1
Carboxylic acid and derivatives 1
(xii) 1,1-Dimethyl-tetrahydro-carboline -3-carboxylic acid
(xiii) 2,7-Dihydroxynapthalene
(xiv) Dihydroxysinapic acid
(xv) Desmethyldiaportinol
(xvi) Allotetrahydroxycortisol
-10
-5
0
5
10
log2 fold change (PDB - GMM)

Response to Editor and Reviewers

Manuscript ID: mSystems00838-26

Corresponding Authors: Paul E. Abraham[1,*] and Tomás A. Rush[1,*]

[1]Biosciences Division, Oak Ridge National Laboratory, Oak Ridge, TN 37831, USA

[*]Corresponding authors - abrahampe@ornl.gov; rushta@ornl.gov

Editor: Dr. Justin van der Hooft

mSystems00838-26 / Decision Letter (1st Revision)

Thank you for the privilege of reviewing your work. Below you will find my comments, instructions from the mSystems editorial office, and the reviewer comments.

The revision answers most of the criticism of the reviewers. Please follow the remaining suggestions before the manuscript can be accepted for publications.

Please return the manuscript within 60 days; if you cannot complete the modification within this time period, please contact me. If you do not wish to modify the manuscript and prefer to submit it to another journal, notify me immediately so that the manuscript may be formally withdrawn from consideration by mSystems.

Dear Dr. van der Hooft,

Thank you for considering a resubmission of our manuscript. We again appreciate the constructive assessment from you and the reviewers.

The minor revisions are as follows:

1. We removed tool-specific reporting from the abstract and formatted Latin phrases for consistency throughout the manuscript.
2. We verified the methodological (https://github.com/dlcarper/Metabolite-Condensing-Script) and data availability (https://doi.org/10.25983/PMI/3366812) links function appropriately.
3. We clarified the limited interpretation and overlap of the brefeldin-like candidate in our dataset, as genomic and spectral agreement remains to be verified.

Below we respond point-by-point.

Editor comment(s)

Comment: The revision answers most of the criticism of the reviewers. Please follow the remaining suggestions before the manuscript can be accepted for publications.

Response: We thank the editor for the opportunity to complete these minor revisions. We have addressed all remaining suggestions in the revised manuscript and respond to each below.

Reviewer 2 comment(s)

Comment 1: Thank you for your efforts and for considering most of our comments. I believe the current manuscript has improved significantly compared to the previous version. It now properly acknowledges the data depth of each individual omic and therefore does not overstate intermediate

findings beyond what the data actually support. Overall, the present study higlights the need for future work on DSEs and makes clear that studying biosynthetic potential and chemical diversity in this group of fungi deserves particular emphasis.

Response: We thank the reviewer for this positive assessment. We are pleased that the revised manuscript now presents the scope and limitations of each omics dataset more clearly and identifies priorities for future work on DSE biosynthetic potential and chemical diversity.

Comment 2: I have added minor comments to the attached document, which can help polish some passages and make some statements a bit more solid, but otherwise I believe the study is a solid contribution to the field and can serve as a nice reference point for future studies.

Response: We thank the reviewer for these additional suggestions. We addressed each annotation as follows.

- Abstract: We removed the highlighted tool-specific statement from the Abstract and checked the formatting of *in silico* and other Latin phrases throughout the main text.
- Brefeldin-like candidate: We re-examined the feature against available brefeldin reference spectra in the GNPS and NIST MS/MS libraries, including spectra linked through the Natural Products Atlas entry for 4-epi-15-epi-brefeldin A (https://www.npatlas.org/explore/compounds/NPA018651). The major fragments support a brefeldin-series scaffold, but the incomplete spectral agreement and low BGC reference similarity do not support a definitive structure-BGC assignment. We therefore revised the Discussion to describe this as a low-confidence brefeldin-like candidate intersection that requires authentic-standard and structural validation.

- Cross-repository spectral comparison: We revised the passage concerning 7-oxoabieta-8,11,13-trien-18-oic acid to distinguish the authentic-standard support for the *H. finlandica* feature from the microbeMASST matches in other taxa. We now clarify that agreement among the major fragments, together with differences among minor peaks, may indicate related diterpenoid scaffolds or structural analogs rather than identical compounds across the bacterial and fungal datasets.
- GCF interpretation: We thank the reviewer for confirming that this cautious interpretation is appropriate. We retained this statement in the revised manuscript.
- Future characterization: We agree and added a concluding statement that these findings warrant targeted chemical and genetic characterization of the secondary metabolite repertoire of *H. finlandica* and other DSEs.

Comment 3: Please make sure all links are openly accesible, such as the GitHub repository, which I could not access, but besides this, it is very nice to see your efforts towards and fair and open data.

Response: We reviewed all repository and data links without an authenticated session. The Metabolite-Condensing-Script GitHub (https://github.com/dlcarper/Metabolite-Condensing-Script) and the public dataset DOI (https://doi.org/10.25983/PMI/3366812) repository are public and can be accessed without signing in. Both links are provided directly in the revised manuscript.

We again thank the editor for inviting our resubmission and reviewers for comments that substantially improved the rigor, framing, and usefulness of the manuscript.

Sincerely,

Paul E. Abraham and Tomás A. Rush

Re: mSystems00838-26R1 (Genome mining and metabolomics reveal context-dependent biosynthetic potential in a core poplar dark septate endophyte)

Dear Dr. Tomas Allen Rush:

Thanks for taking care of the final raised points and suggestions. It is my pleasure to email you that your manuscript has now been accepted for publication in mSystems.
Please note that upon checking myself, the https://www.osti.gov/servlets/purl/3366812 URL did not return any results within 5 minutes. Could you please check again that this is a reliable repository or consider Zenodo as an alternative/additional place to store and make available the supporting data.

Now, I am forwarding it to the ASM production staff for publication. Your paper will first be checked to make sure all elements meet the technical requirements. ASM staff will contact you if anything needs to be revised before copyediting and production can begin. Otherwise, you will be notified when your proofs are ready to be viewed.

Data Availability: ASM policy requires that data be available to the public upon online posting of the article, so please verify all links to sequence records, if present, and make sure that each number retrieves the full record of the data. If a new accession number is not linked or a link is broken, provide production staff with the correct URL for the record. If the accession numbers for new data are not publicly accessible before the expected online posting of the article, publication may be delayed; please contact ASM production staff immediately with the expected release date.

Publication Fees: For information on publication fees and which article types have charges, please visit our website. We have partnered with Copyright Clearance Center (CCC) to collect author charges. If fees apply to your paper, you will receive a message from no-reply@copyright.com with further instructions. For questions related to paying charges through RightsLink, please contact CCC at ASM_Support@copyright.com or toll free at +1-877-622-5543. CCC makes every attempt to respond to all emails within 24 hours.

ASM Membership: Corresponding authors may join or renew ASM membership to obtain discounts on publication fees. Need to upgrade your membership level? Please contact Customer Service at Service@asmusa.org.

Embargo Policy: A press release may be issued as soon as the manuscript is posted on the mSystems Latest Articles webpage. The corresponding author will receive an email with the subject line "ASM Journals Author Services Notification" when the article is available online.

Cover Image Submissions: If you would like to submit a potential Cover Image, please email a file and a short legend to msystems@asmusa.org. Please note that we can only consider images that (i) the authors created or own and (ii) have not been previously published. By submitting, you agree that the image can be used under the same terms as the published article. Image File requirements: TIF/EPS, 7.5 inches wide by 8.25 inches tall (at least 2,250 pixels wide by 2,475 pixels tall), minimum 300 dpi resolution (600 dpi preferred), RGB, and no figure elements, e.g., arrows or panel labels. The legend should be a short description of the image, 1-2 sentences recommended. Please use the ASM Journals Cover Image Sizing Tool (https://journals.asm.org/pb-assets/html/asm-cover-sizing-tool.html) to ensure that your proposed cover image meets our size requirements.

Thank you for submitting your paper to mSystems.

Sincerely,
Justin van der Hooft
Editor
mSystems
