# Supplementary material for: Genome mining and metabolomics reveal context-dependent biosynthetic potential in a core poplar dark septate endophyte
Source: mSystems. 2026 Sep 23;11(10):e00838-26. doi: 10.1128/msystems.00838-26 (PMC13712853; doi:10.1128/msystems.00838-26)
Supplement: Supplementary file 2 [file msystems.00838-26-s0001.docx]

**Supplemental Material S1: Compound discoverer settings used for untargeted metabolomics analysis**

Created with Discoverer version: 3.3.3.200

------------------------------------------------------------------

Processing node: Select Spectra

------------------------------------------------------------------

1. Spectrum Properties Filter:

- Lower RT Limit: 0

- Upper RT Limit: 0

- First Scan: 0

- Last Scan: 0

- Ignore Specified Scans: (not specified)

- Total Intensity Threshold: 0

- Minimum Peak Count: 1

1.1 Spectrum Properties Filter for DDA Spectra:

- Lowest Charge State: 0

- Highest Charge State: 0

- Min. Precursor Mass: 0 Da

- Max. Precursor Mass: 5000 Da

2. Scan Event Filters:

- Mass Analyzer: (not specified)

- Activation Type: (not specified)

- Acquisition Type: (not specified)

- Min. Collision Energy: 0

- Max. Collision Energy: 1000

- Scan Type: Any

- Polarity Mode: Is +

- MS1 Mass Range: (not specified)

- FAIMS CV: (not specified)

3. Peak Filters:

- S/N Threshold (FT-only): 1.5

4. Replacements for Unrecognized Properties:

- Unrecognized Charge Replacements: 1

- Unrecognized Activation Type Replacements: CID

- Unrecognized Polarity Replacements: +

- Unrecognized MS Resolution@200 Replacements: 60000

- Unrecognized MSn Resolution@200 Replacements: 30000

6. General Settings:

- Use Isotope Pattern in Precursor Reevaluation: True

- Store Chromatograms: False

------------------------------------------------------------------

Processing node: Detect Compounds

------------------------------------------------------------------

1. General Settings:

- Mass Tolerance [ppm]: 5 ppm

- Min. Peak Intensity: 10000

- Min. # Scans per Peak: 5

- Use Most Intense Isotope Only: True

- Precursor Mass Tolerance: 0.025 Da

2. Trace Detection:

- Max. Number of Gaps to Correct: 2

- Min. Number of Adjacent Non-Zeros: 2

3. Peak Detection:

- Chromatographic S/N Threshold: 1.5

- Remove Baseline: False

- Gap Ratio Threshold: 0.35

- Max. Peak Width [min]: 1

- Min. Relative Valley Depth: 0.1

4. Isotope Pattern Detection:

- Group Isotopes for: Br; Cl

- RT Tolerance [min]: 0

- Use Peak Quality for Isotope Grouping: True

- Filter out Features with Bad Peaks Only: True

- Zig-Zag Index Threshold: 0.2

- Jaggedness Threshold: 0.4

- Modality Threshold: 0.9

- Remove Potentially False Positive Isotopes: True

5. Compound Assembly:

- Ions: [M+2H]+2; [M+3H]+3; [M+H]+1

- Base Ions: [M+H]+1

- Remove Singlets: True

6. AcquireX Settings:

- Detect Persistent Background Ions: False

------------------------------------------------------------------

Processing node: Group Compounds

------------------------------------------------------------------

1. General Settings:

- Mass Tolerance: 5 ppm

- RT Tolerance [min]: 0.2

- Minimum Valley [%]: 10

- Align Peaks: False

- Preferred Ions: [M+H]+1; [M-H]-1

2. Peak Rating Contributions:

- Area Contribution: 3

- CV Contribution: 10

- FWHM to Base Contribution: 5

- Jaggedness Contribution: 5

- Modality Contribution: 5

- Zig-Zag Index Contribution: 5

3. Peak Rating Filter:

- Peak Rating Threshold: 0

- Number of Files: 0

------------------------------------------------------------------

Processing node: Assign Compound Annotations

------------------------------------------------------------------

1. General Settings:

- Mass Tolerance: 5 ppm

2. Data Sources:

- Data Source #1: mzVault Search

- Data Source #2: (not specified)

- Data Source #3: (not specified)

- Data Source #4: (not specified)

- Data Source #5: (not specified)

- Data Source #6: (not specified)

- Data Source #7: (not specified)

3. Scoring Rules:

- Use mzLogic: True

- Use Spectral Distance: True

- SFit Threshold: 20

- SFit Range: 20

4. Reprocessing:

- Clear Names: False

------------------------------------------------------------------

Processing node: Predict Compositions

------------------------------------------------------------------

1. Prediction Settings:

- Mass Tolerance: 5 ppm

- Min. Element Counts: C H

- Min. RDBE: 0

- Max. RDBE: 40

- Min. H/C: 0.1

- Max. H/C: 3.5

- Max. # Candidates: 10

- Max. # Internal Candidates: 200

2. Pattern Matching:

- Intensity Tolerance [%]: 30

- Intensity Threshold [%]: 0.1

- S/N Threshold: 3

- Min. Spectral Fit [%]: 30

- Min. Pattern Cov. [%]: 90

- Use Dynamic Recalibration: True

3. Fragments Matching:

- Use Fragments Matching: True

- Mass Tolerance: 5 ppm

- S/N Threshold: 3

------------------------------------------------------------------

Processing node: Search mzVault

------------------------------------------------------------------

1. Search Settings:

- mzVault Library: NIST_2023_MSMS_HR1.db|NIST_2023_MSMS_HR2.db|ALL_GNPS.db

- Max. # Results: 10

- Match Factor Threshold: 50

- Search Algorithm: HighChem HighRes

- Match Analyzer Type: False

- IT Fragment Mass Tolerance: 0.4 Da

- FT Fragment Mass Tolerance: 10 ppm

- Use Retention Time: False

- Precursor Mass Tolerance: 10 ppm

- Apply Intensity Threshold: False

- Match Ionization Method: False

- Ion Activation Energy Tolerance: 20

- Match Ion Activation Energy: Match with Tolerance

- Match Ion Activation Type: False

- Remove Precursor Ion: True

- RT Tolerance [min]: 2
