# Supplementary material for: Genome mining and metabolomics reveal context-dependent biosynthetic potential in a core poplar dark septate endophyte
Source: mSystems. 2026 Sep 23;11(10):e00838-26. doi: 10.1128/msystems.00838-26 (PMC13712853; doi:10.1128/msystems.00838-26)
Supplement: Supplementary file 3 [file msystems.00838-26-s0003.docx]

**Supplemental Figure S1.** microbeMASST search result (left) for (**A**) 7-oxoabieta-8,11,13-trien-18-oic acid, (**B**) Desmethyldiaportinol, and (**C**) desferricrocin observed in our dataset compared to other publicly available deposited samples and (right) corresponding spectral matches. The number above each bar represents the number (**A-B**) and percentage (**C**) of respective matches to the query spectrum.
