# Supplementary material for: Genome mining and metabolomics reveal context-dependent biosynthetic potential in a core poplar dark septate endophyte
Source: mSystems. 2026 Sep 23;11(10):e00838-26. doi: 10.1128/msystems.00838-26 (PMC13712853; doi:10.1128/msystems.00838-26)
Supplement: Supplementary file 4 [file msystems.00838-26-s0004.docx]

**Supplemental Figure S2.** Spectral matches to query spectrum using LC MS/MS. (**A**) (top) m/z 193.0495 corresponding to Dihydroxynaphthalene observed in *Hyaloscypha finlandica* spent media and (bottom) m/z 193.0495 corresponding to 1,3,6,8-Tetrahydroxynaphthalene (i.e. a melanin pathway intermediate; CAS 18512-30-6) from commercially purchased standard. (**B**) Mirror match for 7-oxodehydroabietic acid observed in *H. finlandica* spent media (top) and commercially produced and purchased standard (bottom).
